# Stem cell secretome treatment improves whole‐body metabolism, reduces adiposity, and promotes skeletal muscle function in aged mice

**DOI:** 10.1111/acel.14144

**Published:** 2024-03-18

**Authors:** Zachary J. Fennel, Paul‐Emile Bourrant, Anu Susan Kurian, Jonathan J. Petrocelli, Naomi M. M. P. de Hart, Elena M. Yee, Sihem Boudina, Hans S. Keirstead, Gabrielle Nistor, Scott A. Greilach, Nicole C. Berchtold, Thomas E. Lane, Micah J. Drummond

**Affiliations:** ^1^ Department of Physical Therapy and Athletic Training University of Utah Salt Lake City Utah USA; ^2^ Division of Nutrition and Integrative Physiology University of Utah Salt Lake City Utah USA; ^3^ Immunis, Inc. Irvine California USA; ^4^ Department of Neurobiology and Behavior University of California Irvine California USA; ^5^ Molecular Medicine Program University of Utah Salt Lake City Utah USA

**Keywords:** adipogenesis, fibrosis, lipids, metabolic rate, sarcopenia, stem cells

## Abstract

Aging coincides with the progressive loss of muscle mass and strength, increased adiposity, and diminished physical function. Accordingly, interventions aimed at improving muscle, metabolic, and/or physical health are of interest to mitigate the adverse effects of aging. In this study, we tested a stem cell secretome product, which contains extracellular vesicles and growth, cytoskeletal remodeling, and immunomodulatory factors. We examined the effects of 4 weeks of 2×/week unilateral intramuscular secretome injections (quadriceps) in ambulatory aged male C57BL/6 mice (22–24 months) compared to saline‐injected aged‐matched controls. Secretome delivery substantially increased whole‐body lean mass and decreased fat mass, corresponding to higher myofiber cross‐sectional area and smaller adipocyte size, respectively. Secretome‐treated mice also had greater whole‐body physical function (grip strength and rotarod performance) and had higher energy expenditure and physical activity levels compared to control mice. Furthermore, secretome‐treated mice had greater skeletal muscle Pax7+ cell abundance, capillary density, collagen IV turnover, reduced intramuscular lipids, and greater Akt and hormone sensitive lipase phosphorylation in adipose tissue. Finally, secretome treatment in vitro directly enhanced muscle cell growth and IL‐6 production, and in adipocytes, it reduced lipid content and improved insulin sensitivity. Moreover, indirect treatment with secretome‐treated myotube culture media also enhanced muscle cell growth and adipocyte size reduction. Together, these data suggest that intramuscular treatment with a stem cell secretome improves whole‐body metabolism, physical function, and remodels skeletal muscle and adipose tissue in aged mice.

## Graphical abstract

In this study, we show that the 4‐week administration of a stem cell secretome, made up of exosomes, growth, immune, and extracellular matrix factors, robustly ameliorated several hallmarks of aging in male mice resulting in improvements in physical function, metabolism, adiposity, and skeletal muscle remodeling.

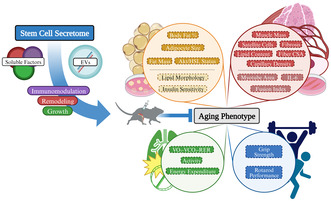

**Abbreviations**
%: percent
4E‐BP1: eukaryotic translation initiation factor 4E binding protein
Akt: protein kinase B
B‐CHP: collagen hybridizing peptide
CD31: cluster of differentiation 31
Cer: ceramide
CLAMS: Columbus Instruments Comprehensive Lab Animal Monitoring System
CM: cultured media
COL‐IV: collagen IV
CSA: cross‐sectional area
DAG: diglyceride
DAPI: 4’,6‐diamidino‐2‐phenylindole
ERK1/2: extracellular signal‐regulated kinase 1/2
E‐WAT: epididymal fat pad
FBXO32: f‐box protein 32
FOXO3a: forkhead box O3
GTT: glucose tolerance test
h: Hours
H&E: hematoxylin and eosin
HOMA‐IR: homeostatic model assessment for insulin resistance
HSl: hormone sensitive lipase
IF: immunofluorescence
IL‐6: interleukin 6
I‐WAT: inguinal fat pad
min: minutes
mTOR: mechanistic target of rapamycin
MUSA1: f‐box protein 30
MyHC: myosin heavy chain
NMR: nuclear magnetic resonance
OCT: optimal cutting temperature compound
P/T: Phospho‐total
Pax7: paired box protein pax‐7
PGC‐1α: peroxisome proliferator‐activated receptor‐gamma coactivator 1 alpha
qPCR: real‐time polymerase chain reaction
RER: respiratory exchange ratio
RNA: ribonucleic acid
rps6k: ribosomal binding protein S6 kinase B1
TAG: triglyceride
Traf6: tumor necrosis factor receptor associated factor 6
USP: United States pharmaceutical
VCO_2_
: carbon dioxide production
VO_2_
: oxygen consumption

## 1 INTRODUCTION

Aging is accompanied by the progressive development of frailty, chronic diseases, and impaired quality of life. While multifaceted, the severity of the aging phenotype is largely controlled by musculoskeletal and metabolic health and associated cellular and molecular underpinnings (Fried et al., 2021; Kennedy et al., 2014). Skeletal mass and function as well as metabolic health are markedly impaired in older adults and are associated with numerous negative health effects under the umbrella of aging (Larsson et al., 2019; Pontzer et al., 2021). Aged skeletal muscle is characterized by structural disorganization (Wood et al., 2014), fibrosis, neuromuscular degeneration (Liu et al., 2017), blunted growth, and impaired regenerative capacity (Tidball et al., 2021). Similarly, adipose tissues exhibit metabolic dysregulation (Goodpaster et al., 2005), adverse redistribution (Goodpaster et al., 2005), and inflammatory profiles in aged individuals (Wang et al., 2022). Moreover, aging‐induced muscle and adipose dysfunction, either individually or combined (termed sarcopenic obesity) (Trouwborst et al., 2018), diminish the effectiveness of exercise and dietary interventions (Peterson et al., 2011; Phillips et al., 2017) and lead to generalized frailty, increased disease morbidity, and ultimately, pre‐mature mortality (Fried et al., 2021; He et al., 2021; Kennedy et al., 2014). Therefore, there is great interest in the development of therapeutic strategies that target age‐related deficits in musculoskeletal and adipose tissue health that result in improved physical activity and physical function levels (Chakhtoura et al., 2023; Kwak & Kwon, 2019).

Stem cell therapies show promise in regenerative medicine, yet when delivered directly may induce adverse responses. However, secondary approaches have arisen including the enrichment of culture media with stem cell secretory factors (Fix et al., 2021; Kim et al., 2016; Sandonà et al., 2021; Sanz‐Ros et al., 2022; Wu et al., 2022). These secretomes include numerous soluble and encapsulated (extracellular vesicles) signaling molecules (cytokines, growth factors) that exert cellular and tissue adaptive effects (Daneshmandi et al., 2020; Hsieh et al., 2013; Sandonà et al., 2021). Suitably, secretome products from various stem cell origins have been utilized to enhance muscular outcomes and combat the effects of aging in mice (Sanz‐Ros et al., 2022), including use of these products in a regenerative capacity following muscular atrophy or damage (Amankwaah et al., 2019; Fix et al., 2021; Wu et al., 2022). For example, we previously demonstrated that intramuscular secretome treatment reduces the loss of muscle mass and strength associated with disuse‐atrophy and accelerates recovery from hindlimb unloading‐associated atrophy (Fix et al., 2021). Similarly, others have shown that intravenous treatment with extracellular vesicles from adipocyte‐derived stem cells improves muscular strength and function and decreases frailty in old mice (Sanz‐Ros et al., 2022). Ostensibly, stem cell secretome treatments can combat the effects of aging in skeletal muscle, yet the secondary whole‐body effects including changes in adipose tissue and body composition, as well as metabolic function are unknown.

Therefore, the purpose of this study was to investigate the effects of twice weekly intramuscular treatment for 4 weeks with a pluripotent stem cell secretome product in aged mice on whole‐body metabolism (energy expenditure, tissue composition, activity levels), physical function, as well as skeletal muscle and adipose tissue remodeling. Furthermore, we examined the acute skeletal muscle transcriptional response to the secretome treatment as well as the autocrine and paracrine effects of the secretome product on muscle cells and adipocytes in‐vitro. We hypothesized that chronic intramuscular secretome treatment would ameliorate muscle aging (decrease muscle fibrosis, increase capillarity, stem cells, and myofiber hypertrophy) and adiposity while improving whole‐body energy metabolism and physical function capacity in aged mice. Moreover, we hypothesized that acute secretome treatment would directly and indirectly impact C2C12 muscle cell growth and reduce lipid accumulation in 3T3‐L1 adipocytes.

## 2 METHODS

### 2.1 Animals and experimental design

Male C57BL/6 mice (obtained from the National Institute of Aging rodent colony) began experiments at 22–24 months and finished at 23–25 months. Mice were maintained in a temperature‐controlled (22–23°C) facility on a 12:12‐h light/dark cycle and housed with ad libitum food and water access. After ≥1 week of acclimating to their cages, mice were assigned to either secretome treatment or saline control groups (*n* = 16/group) in a matched fashion (body, fat, and lean mass). After the intervention, a subset of mice (*n* = 6/group) underwent metabolic chamber measurements (detailed below). These same mice were utilized to examine the effects of secretome/saline treatment and withdrawal for 2 weeks after the final injections. Most of the remaining mice (*n* = 8/group) were fasted for ~4 h and euthanized under isoflurane followed by cervical dislocation. Tissues (quadriceps, gastrocnemius, inguinal: I‐WAT and epididymal: E‐WAT fat pads, heart, liver) were dissected, weighed, and placed in paraformaldehyde, frozen in liquid nitrogen, or frozen in optimal cutting temperature compound (OCT) (Fisher Scientific 23‐730‐571, Waltham, MA, USA) in isopentane, and stored at −80°C based on the specific analysis. All animal procedures were conducted in agreement with standards set by the University of Utah Institutional Animal Care and Use Committee.

### 2.2 Secretome treatment

The secretome treatment was derived from partially differentiated pluripotent human embryonic stem cells (CSC14, CVCL_B918). Cultured media collected from these cells was pooled, sterile filtered, concentrated, and prepared as a USP‐grade cell‐free stem cell‐based secretome product (IMMUNA; Immunis, Inc., Irvine, CA). Fifty microliters of secretome or saline (0.9% USP) were delivered to the right quadricep muscle via intramuscular injection under sterile conditions twice per week for 4 weeks (8 total injections/mouse). Secretome treatment was injected at a 0.4% concentration in saline based on pilot data and our previous work (Fix et al., 2021).

As previously described by our lab (Fix et al., 2021), the secretome product contains a host of soluble signaling molecules with prominent proteins regulating cellular growth, remodeling, and immunomodulation (Table 1). Extracellular vesicles from the secretome product were examined via nanoparticle tracking followed by microRNA isolation and next‐generation sequencing by an independent party (Creative Biolabs, Inc.). Extracellular vesicles had an average particle size of 118 (nm) and concentration 2.8^12^ (particles/mL), while microRNA concentration was 23 ng/μL with a quality score of 30 (99.9%). The top 10 identified microRNAs and their validated target genes are also presented in Table 1.

**TABLE 1:** Secretome factors.

Growth factors	Fetuin A; Periostin; albumin; TFPI; VEGF R1; Midkine; Pref‐1; IGFBP‐6; Follistatin; IGFBP‐6; ErbB3; GDF‐15; FGF‐1; FGF‐2; insulin; Angiogenin; TGFb1; Angiostatin; ANG‐1.
Remodeling factors	MMP‐9; P‐Cadherin; Nidogen‐1; EMMPRIN; Contactin‐2; TIMP‐1; Legumain; Syndecan‐4; CF XIV; Cathespsin L; BCAM; Syndecan‐1; ICAM‐1; Cathepsin B; Serpin A4; Clusterin; EpCAM; E‐Cadherin; Kallikrein 5.
Immune factors	CD48 Ferritin; PAI‐1; MIF; OPN; GROa; ADAM8; Crystatin B; B2M; Galectin‐1; CXCL16; B7‐H3; GRO; MCP‐1.
Extracellular vesicle‐Contained miRNAs	miR‐93‐5p; miR‐335‐5p; miR‐302d‐3p; miR‐302a‐5p; miR‐302a‐3p; miR‐20b‐5p; miR‐20a‐5p; miR‐16‐2‐5p; miR‐16‐1‐5p; miR‐130a‐3p.
miRNA Gene Targets	CSF1; MAFB; MEOX2; HOXA5; TAC1; ATXN1; DDX6; TPPP3; BCL2; VEGFA.
Soluble factors & top 10 microRNAs (miRNAs) and their gene targets identified from within the secretome treatment categorized by generalized function or as the top 10 most prevalent, respectively. Soluble factor results derived via ELISA analysis as reported by Fix et al. (2021), miRNAs (presented as precursor) and gene targets via nanoparticle tracking and next generation sequencing.	

Abbreviations: ADAM8, A disintigren and metalloproteinase domain‐containing protein 8; ANG‐1, angiopoietin 1; ATXN1, ataxin 1; B2M, beta‐2 microglobulin; B7‐H3, B7 homolog 3; BCAM, basal cell adhesion molecule; BCL2, B‐cell lymphoma 2 apoptosis regulator; CD48, cluster of differentiation 48; CSF1, colony stimulating factor 1; CXCL16, chemokine ligand 16; DDX6, DEAD‐box helicase 6; EMMPRIN, extracellular matrix metalloproteinase inducer; EpCAM, epithelial cellular adhesion molecule; GROa, chemokine ligand 1; HOXA5, homeobox A5; ICAM‐1, intercellular adhesion molecule 1; IGFBP‐6, insulin like growth factor binding protein 6; MAFB, V‐maf musculoaponeurotic fibrosarcoma oncogene homolog B; MCP‐1, monocyte chemoattractant protein‐1; MEOX2, mesenchyme homeobox 2; MIF, macrophage migration inhibitory factor; MMP‐9, matrix metalloproteinase‐9; OPN, osteopontin; PAI‐1, plasminogen activator inhibitor‐1; pref‐1, preadipocyte secreted factor 1; TAC1, tachykinin precursor 1; TFPI, tissue factor pathway inhibitor; TGFb1, transforming growth factor beta 1; TIMP‐1, tissue inhibitor of metalloproteinase 1; TPPP3, tubulin polymerization promoting protein family member 3; VEGF R1, vascular endothelial growth factor receptor 1; VEGFA, vascular endothelial growth factor A.

### 2.3 Body tissue composition and physical function testing

A nuclear magnetic resonance (NMR) instrument (Bruker Minispec MQ20 NMR analyzer, Rheinstetten, Germany) was used to assess whole body tissue composition. In addition, whole‐body strength, plus balance and coordination, were assessed by grip strength and rotarod instrument, respectively, as we have conducted previously (Petrocelli et al., 2021). NMR and grip strength were assessed weekly, while rotarod was tested before treatment and then repeated after the 4‐week intervention. Whole‐body grip strength was assessed using a grip strength meter with a mesh wire attachment (Columbus Instruments, Columbus, OH, USA). After acclimation testing a week prior, mice were placed on the mesh wire and pulled by the base of their tail, parallel to the mesh wire. Peak force was recorded, and an average of three trials was recorded. Balance and coordination were assessed using rotarod testing on a Rotamex‐5 (Columbus Instruments, Columbus, OH). The speed began at 0.1 rpm and increased by 0.3 rpm/s increments with the final recorded time when mice fell off the rotating bar. Each mouse performed the test three times, and an average time was recorded. Mice were acclimated on the rotarod 2 days prior to testing. All physical function tests were conducted by the same research personnel.

### 2.4 Metabolic measurements

To measure whole‐body metabolic parameters, a subset of mice were placed in metabolic cages (CLAMS; Columbus Instruments Comprehensive Lab Animal Monitoring System (Columbus, OH, USA, serial# 180072)) for 72 h at the end of the 4‐week experimental intervention. Mice were single‐housed and acclimated for 48 h with the final 24 h data used for analysis. Respiratory exchange ratio (RER) was calculated from V̇CO_2_ production and V̇O_2_ consumption, and energy expenditure was calculated by dividing heat production (kcal/hr) by body weight. Ambulatory activity was calculated by summing ambulatory beam breaks in the x, y, and z directions. Food intake was calculated from a food scale inside the CLAMS unit.

Prior to tissue collection following the 4‐week experiments, separate mice from above performed a 120‐min glucose tolerance test (10% glucose solution injected at 1 g/kg) with tail blood sampling including assessment of fasting and 30 min fed insulin levels via Ultra‐Sensitive Mouse ELISA kit (Crystal Chem, Elk Grove Village, Il, USA) per manufacturer recommendations.

### 2.5 Immunohistochemistry

Frozen OCT‐embedded quadriceps (cut in a longitudinal plane) were sectioned at a thickness of 10 μm using a Leica cryostat (CM1860, Lecia Biosystems, Wetzlar, DE) and maintained at −20°C until stained. Muscle sections were stained to assess myofiber cross‐sectional area (CSA) and myosin heavy chain (MyHC) fiber type, satellite/muscle stem cell content (Pax7+), capillarization (CD31+), and collagen IV turnover as described previously (Fix et al., 2021; Petrocelli et al., 2021). Detailed methodologies and reagents are reported in the Supplemental Methods.

### 2.6 Fat and liver histology

After dissection, a portion of each fat pad and the left lobe of the liver were placed in 4% paraformaldehyde for 24 h, after which they were stored in 70% ethanol until analysis. Samples were submitted to Associated Regional and University Pathologists (ARUP) laboratories at the University of Utah and the Department of Pathology for hematoxylin and eosin (H&E) (fat pads) while livers were stained for H&E and Masson's trichome. Briefly, samples were embedded in paraffin, sectioned at 5μm thickness then H&E stained to visualize lipid droplets, and trichrome stained to visualize tissue fibrosis. Slides were imaged on a Zeiss Slide Scanner Axio Scan.Z1 (Carl Zeiss Inc.) with a 10× (fat pads, liver trichrome) or 20× (liver H&E) objective lens. Fat pad images were analyzed using a Fiji plugin Adiposoft as described by others (Galarraga et al., 2012) to determine average adipocyte diameter for each sample across three randomly selected fields. Liver lipid accumulation was assessed from H&E images in Fiji, briefly, by thresholding to identify lipid droplets, analyzing particles, and removing erroneous areas from analysis. Liver fibrosis was analyzed using the Automated Fibrosis Analysis Toolkit plugin for Fiji as described elsewhere (Gratz et al., 2020).

### 2.7 RNA sequencing and Western blotting

In a separate experiment to determine the acute effects of the secretome treatment on muscle, we delivered a single intramuscular treatment of secretome or saline to C57BL/6 mice (26–28 months old) in a fasted (4 h) state. Three hours after injection, mice were euthanized, and injected quadriceps were collected for bulk RNA sequencing, qPCR, and immunoblotting. Hallmark, KEGG, and REACTOME pathways were identified using the fast gene set enrichment analysis in MSigDB using a 5% FDR. Data can be found on the Gene Expression Omnibus (GSE242211).

Injected quadriceps muscle from the acute and 4‐week experiments as well as adipose pads from the 4‐week investigation were additionally used to isolate RNA and/or protein for downstream real‐time PCR and western blotting for anabolic, catabolic, and metabolic targets. Additional details on methodology can be found in the Supplemental Methods.

### 2.8 Lipidomics

A portion of injected quadricep muscle (~15 mg) from the 4‐week experimental study was used to extract lipids and prepare samples for liquid chromatography mass spectrometry (LC–MS) metabolomic analysis as described elsewhere (de Hart et al., 2023). Processed lipid metabolites including triglycerides, ceramides, and diacylglycerides with relative standard deviation less than 30% of quality controls were examined individually and as population totals. Detailed methods for all analyses can be found in the Supplemental Methods.

### 2.9 Adipocyte and muscle cell culture experiments

C2C12 myoblasts were grown to confluence then differentiated for ~4 days under standard conditions in 6‐well dishes. Media was replaced every 48 h during growth and differentiation. Cultured myotubes were treated with 4% secretome product replacement for 24 h based on previous experiments (Fix et al., 2021) and analyzed for changes to myotube area and fusion index (*n* = 6). Separate groups of control and treated (4% secretome) myotubes were used to produce culture media for 3 h (*n* = 6). Culture media was then placed on fully differentiated myotubes for 24 h to assess autocrine and paracrine effects of cultured media followed by the same measurements described above (*n* = 4). Cultured media replicates (*n* = 7) and undiluted secretome product (*n* = 3) were additionally tested for IL‐6 concentration using a Mouse Quantikine ELISA Kit per manufacturer recommendations (R&D Sytems, Minneapolis, MN, USA). Detailed methodologies and reagents can be found in the Supplemental Methods.

3T3‐L1 preadipocytes were prepared according to manufacturer recommendations then differentiated into adipocytes in 8‐well chamber slides for 5–7 days until confluent, followed by 3 days of differentiation, and finally cellular maintenance and treatment (Montanari et al., 2019). Media was replaced every 48–72 h during growth and differentiation. Replicate wells of cultured adipocytes (*n* = 7) were treated via 5% or 20% secretome product media replacement for 24 h and compared to untreated controls. Adipocytes were stained and assessed for content as described elsewhere (Montanari et al., 2019) and in the Supplemental Methods. Separate groups (*n* = 6) of control and treated adipocytes (20% secretome) with and without insulin treatment (100 nM) were then analyzed for insulin sensitivity via western blot (Akt phosphorylation, Ser473). Finally, groups of differentiated 3T3‐L1 adipocytes (*n* = 4) were treated with 20% media replacement using the C2C12 culture mediums above and assessed as previously described.

### 2.10 Statistical analyses

All data are shown as mean ± SD. Where appropriate, one‐way or two‐way ANOVAs were used with Holm‐Bonferroni or Dunnett's multiple comparisons test. If missing data points were present or groups were unbalanced, mixed effects analysis was used in place of ANOVA. When comparing two groups at a single time point, unpaired and paired *t*‐test were used where appropriate. Data sets were assessed visually for normality via Q–Q plots, skewedness, and kurtosis, and tested with Shapiro‐Wilks test if necessary. Statistical significance was set to *p* < 0.05. GraphPad Prism (v10.0.1, La Jolla, CA, USA) was utilized for all statistical analyses and figure assembly.

## 3 RESULTS

### 3.1 Secretome treatment enhanced whole‐body metabolic rate and physical activity levels in aged mice

**FIGURE 1:**
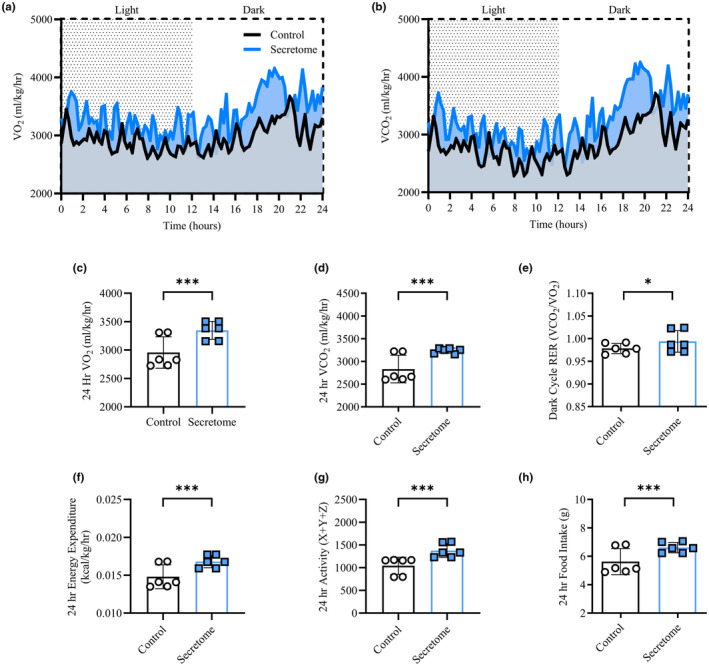
Whole‐Body Metabolic Profile. Oxygen consumption (VO_2_ – ml/kg/hr) and carbon dioxide production (VCO_2_ – ml/kg/hr) across 24 h in comprehensive lab animal monitoring system (CLAMS) for control and secretome‐treated mice (a, b). 24‐h average VO_2_ (c) and VCO_2_ (d), 12‐h average dark cycle respiratory exchange ratio (RER – VCO_2_/VO_2_; e), 24‐h average energy expenditure (f; kcal/kg/hr), activity (g; movement – X + Y + Z), and food intake (g) (h). All data presented as mean ± SD, white bars represent controls while blue squares represent secretome‐treated mice. *n* = 6 for both groups. * indicates significant difference between conditions for the indicated timepoints with * = *p* < 0.05, ** = *p* < 0.01, and *** = *p* < 0.001.

**FIGURE 2:**
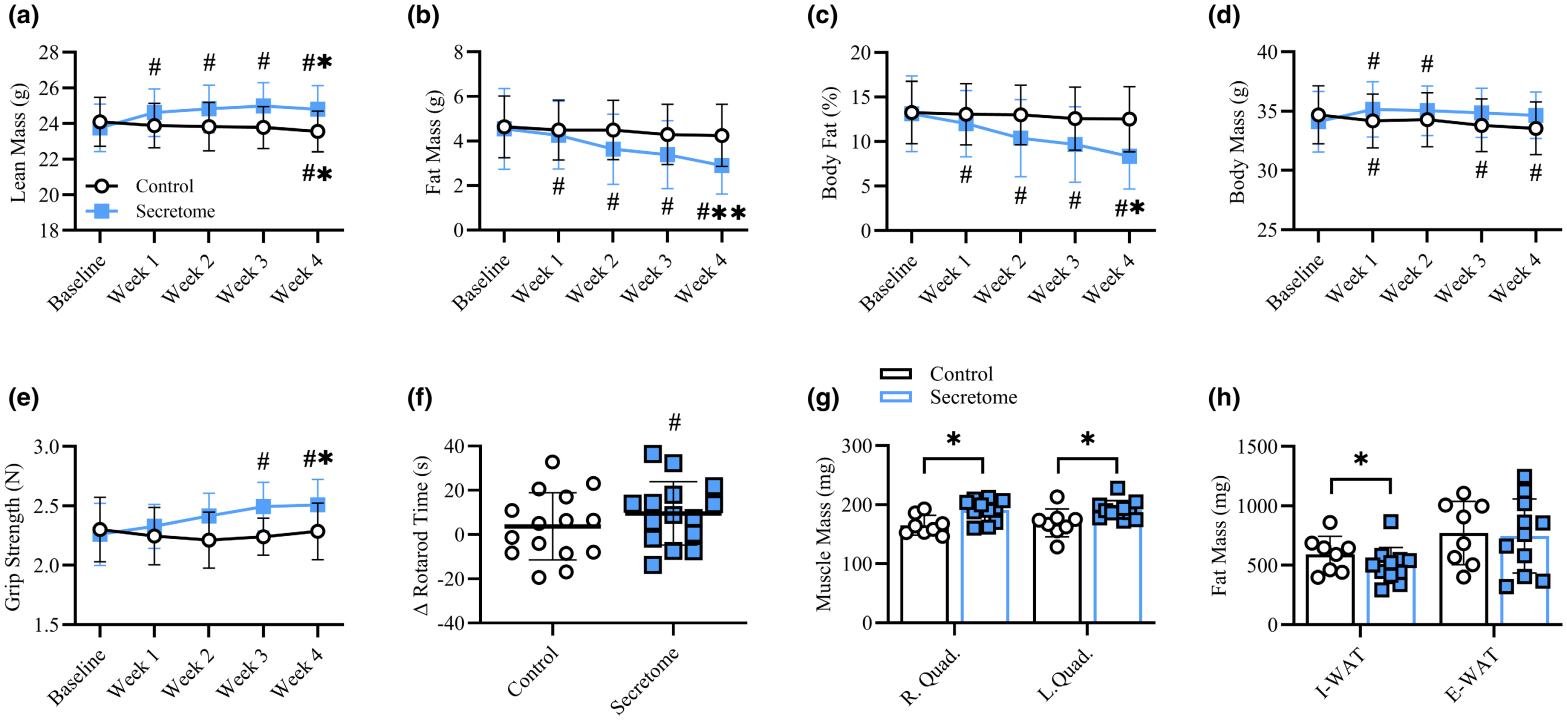
Whole body Tissue and Physical Function Changes. Weekly changes lean mass (a), fat mass (b), body fat % (c), and body mass (d) for control and secretome‐treated mice. Weekly whole‐body grip strength (e) and change (Δ) in rotarod performance time from baseline (f). Quadriceps mass (g) and fat mass (h) following 4‐weeks. Control mice are represented by black circles and bars while secretome‐treated mice are noted by blue squares and bars. All data presented as mean ± SEM. (a–h) control mice (*n* = 15), secretome‐treated mice (*n* = 16). Weekly changes analyzed via mixed‐effects model with Holm‐Bonferroni multiple comparisons plus planned comparison *t*‐tests at the 4‐week timepoint. # indicates significant difference (*p* < 0.05) from baseline for respective group. * indicates significant difference (*p* < 0.05) between groups at the indicated time point.

To explore the whole‐body metabolic effects of secretome treatment on aged mice, we performed 24 h of metabolic profiling following 4 weeks of secretome treatment versus saline using CLAMS (Figure 1a, b). We found that secretome‐treated mice displayed greater oxygen consumption (Figure 1c; 3347 ± 353 vs. 2957 ± 233 mL/kg/hr, *p* < 0.01), CO_2_ production (Figure 1d; 3237 ± 440 vs. 2832 ± 312 mL/kg/hr, *p* < 0.01), and dark cycle respiratory exchange ratio (Figure 1e; *p* = 0.02) compared to control mice. Accordingly, 24‐h energy expenditure (Figure 1f; 0.016 ± 0.0 vs. 0.014 ± 0.0, kcal/kg/hr *p* < 0.01), physical activity levels (Figure 1g; 1377 ± 603 vs. 1041 ± 369 X + Y + Z, *p* < 0.01), and food intake (Figure 2h; 6.6 ± 1.3 vs 5.6 ± 1.2 g, *p* < 0.01) were significantly elevated in secretome‐treated compared to control mice. Following 4‐weeks of secretome treatment, mice did not have different fasting glucose (83.1 ± 9.5 vs. 90.0 ± 10.0 mg/dL, *p* = 0.70) or responses to glucose stimulation following an i.p. glucose tolerance test measured by area under the curve analysis (150.3 ± 52.8 vs. 143.8 ± 41.7 au, *p* = 0.81) compared to control mice. Similarly, insulin levels were not different at fasting (0.55 ± 0.13 vs. 0.46 ± 0.06 ng/mL, *p* = 0.20) or following glucose injection (0.97 ± 0.16 vs. 0.98 ± 0.07 ng/mL, *p* = 0.96) comparing secretome‐treated and control mice. Moreover, HOMA‐IR (fasting insulin × fasting glucose) was not different between the groups (2.0 ± 0.6 vs. 1.8 ± 0.2 au, *p* = 0.46).

### 3.2 Secretome treatment increased lean mass and physical function and reduced whole body fat mass in aged mice

To gain insight into the whole‐body metabolic effects of the secretome treatment, we analyzed the weekly effects of the secretome treatment on whole‐body tissue content via NMR spectroscopy, the impact on physical function (grip strength and rotarod performance) at baseline and at the end of the 4‐week treatment, and localized effects on specific tissues (quadriceps, I‐WAT, E‐WAT). Weekly assessment revealed that the secretome treatment progressively increased body mass and decreased body fat percentage over baseline levels, which were driven by consistent increases in lean mass and decreases in fat mass (Figure 2a–d). Conversely, aged mice treated with saline showed a decrease in body mass compared to baseline (Figure 2d), likely as result of modest decrements in lean mass (Figure 2a) by week 4. Group comparison after 4 weeks treatment demonstrated greater lean mass (24.8 ± 1.3 vs. 23.6 ± 1.1 g, *p* = 0.02), lower fat mass (2.9 ± 1.3 vs. 4.3 ± 1.4 g, *p* = 0.02), and lower body fat percentage (12.5 ± 3.7 vs. 8.4 ± 3.7%, *p* < 0.01) in secretome‐treated mice relative to control mice (Figure 2a–c). Assessment of strength, balance, and coordination demonstrated that (1) whole‐body grip strength was increased over baseline strength at 3 and 4 weeks in secretome‐treated mice; (2) secretome‐treated mice were significantly stronger than control mice at the 4‐week timepoint (2.5 ± 0.2 vs. 2.3 ± 0.3 N, *p* = 0.03; Figure 2e); and (3) secretome‐treated mice, but not saline‐treated controls, had increased rotarod performance time compared to baseline performance (Δ9.5 ± 14.3 sec, *p* = 0.03; Figure 2f). Individual values for all described measures at baseline and Week 4 are presented in Figure S1A–D. Finally, though we could not fully characterize frailty index (Baumann et al., 2020), we noted a greater proportion of control versus the secretome‐treated mice were below or equal to the 50th (62.5% vs. 33.3%) and 25th (37.5% vs. 6.7%) percentiles of the combined population considering the weight loss, weakness, and walking speed categories (data not shown).

To assess persistence of the described whole‐body tissue composition and physical function effects, in a separate group of mice, we ceased the secretome treatment for 2 weeks (withdrawal) following the 4‐week treatment period and compared this response to the 4‐week treatment timepoint for whole body tissue composition outcomes, grip strength, rotarod performance, and tissue weights (Figure S1). We found that following 2 weeks of withdrawal in previously secretome‐treated mice, that the prior gains in body mass (Figure S1E) and lean mass (Figure S1F) were lost. However, secretome‐mediated adaptations in fat mass, % body fat, grip strength, and rotarod performance (Figure S1G–J) were maintained.

Next, we assessed the localized effects of secretome treatment on specific tissues (quadriceps, I‐WAT, and E‐WAT). After 4 weeks, secretome‐treated mice had greater right (190.8 ± 18.9 vs. 165.3 ± 16.7 mg, *p* < 0.01) and left (191.0 ± 15.9 vs. 169.2 ± 23.7 mg, *p* = 0.02) quadriceps mass compared to control mice (Figure 2g). Furthermore, secretome‐treated mice had lower I‐WAT (462.0 ± 103.4 vs. 591.7 ± 152.9 mg, *p* = 0.03) compared to control mice, while E‐WAT did not differ (Figure 2h). Finally, gastrocnemius, liver, and heart weights were not different between groups at 4‐weeks (*p* > 0.05; Table S1).

### 3.3 Secretome treatment increased myofiber size and promoted myofiber remodeling and transcriptional reprogramming in aged mice

**FIGURE 3:**
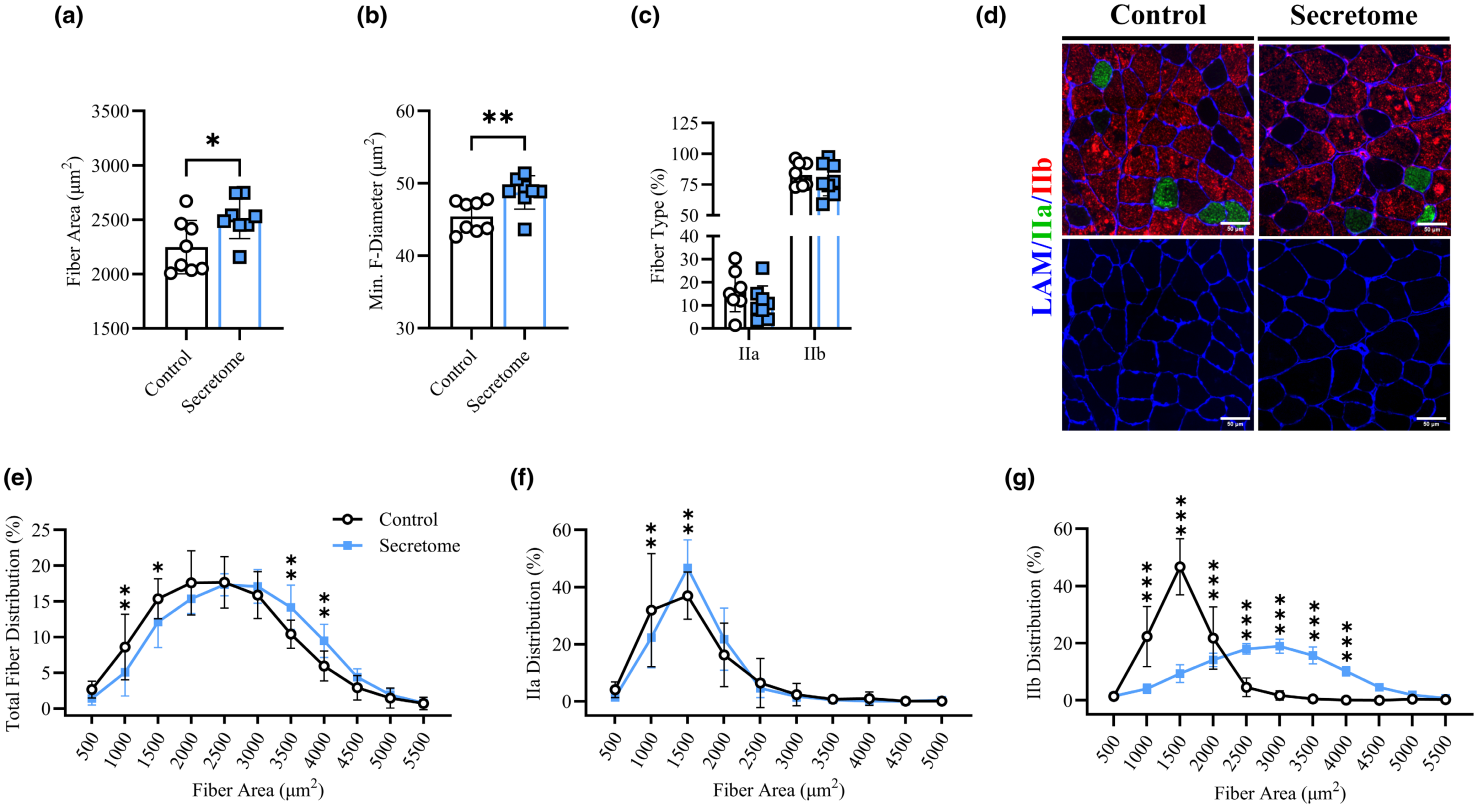
Skeletal Muscle Morphology. Average quadriceps fiber cross sectional area μm^2^ (a), minimum feret diameter μm^2^ (b), and fiber type proportion (c; MyHC – IIa/IIb %) for control (*n* = 8) and secretome treated (*n* = 8) mice. Size distribution (%) for total (e), IIa (f) and IIb (g) fiber types across 500 μm^2^ increments. Representative histochemical image of fiber type including cell border with laminin in blue, MyHC IIa in green, MyHC IIb in red, and scale bar of 50 μm (d). All data presented as mean ± SD, white circles and bars represent controls while blue squares and bars represent secretome‐treated mice. *n* = 8 for each group. Analyzed via *t*‐tests and mixed effects models with Holm‐Bonferroni comparisons. * indicates significant difference between groups at indicated category with * = *p* < 0.05, ** = *p* < 0.01, *** = *p* < 0.001.

We next used immunohistochemistry to analyze the effects of 4‐week‐secretome treatment on quadricep muscle morphology (CSA, feret diameter, fiber type) and cellular remodeling events (satellite cells, capillarization, collagen IV turnover), physiological parameters that are notably disrupted in muscle aging (Alnaqeeb et al., 1984; Lacraz et al., 2015; Zimmerman et al., 1993). After 4‐weeks of secretome treatment, mice had greater average myofiber CSA (Figure 3a; 2514 ± 186.9 vs. 2248 ± 245.4 μm^2^, *p* = 0.03) and minimum feret diameter (Figure 3b; 48.7 ± 2.3 vs. 45.4 ± 2.2 μm^2^, *p* = 0.01) compared to saline‐treated controls. Fiber type % (MyHC IIa and IIb) was not different between groups (Figure 3c), nor was fiber size altered based on fiber type (data not shown). However, secretome‐treated quadriceps displayed a rightward shift in the size distribution of total IIa and IIb fibers (Figure 3e–g). Notably, analysis by 500 μm^2^ increments demonstrated that the proportion of total fibers (Figure 3e) >500–1500 μm^2^ was lower while fibers >3500–4000 μm^2^ were greater comparing secretome‐treated to control mice. Additionally, there were less IIa fibers (Figure 3f) between >500 and 1000 μm^2^ and more fibers >1000–1500 μm^2^ in secretome‐treated muscles. Similarly, secretome‐treated mice displayed a lower proportion of IIb fibers (Figure 3g) >500–1500 μm^2^ and a higher proportion >2500–4000 μm^2^ compared to controls. Representative histochemical images of fiber types are shown in Figure 3d.

**FIGURE 4:**
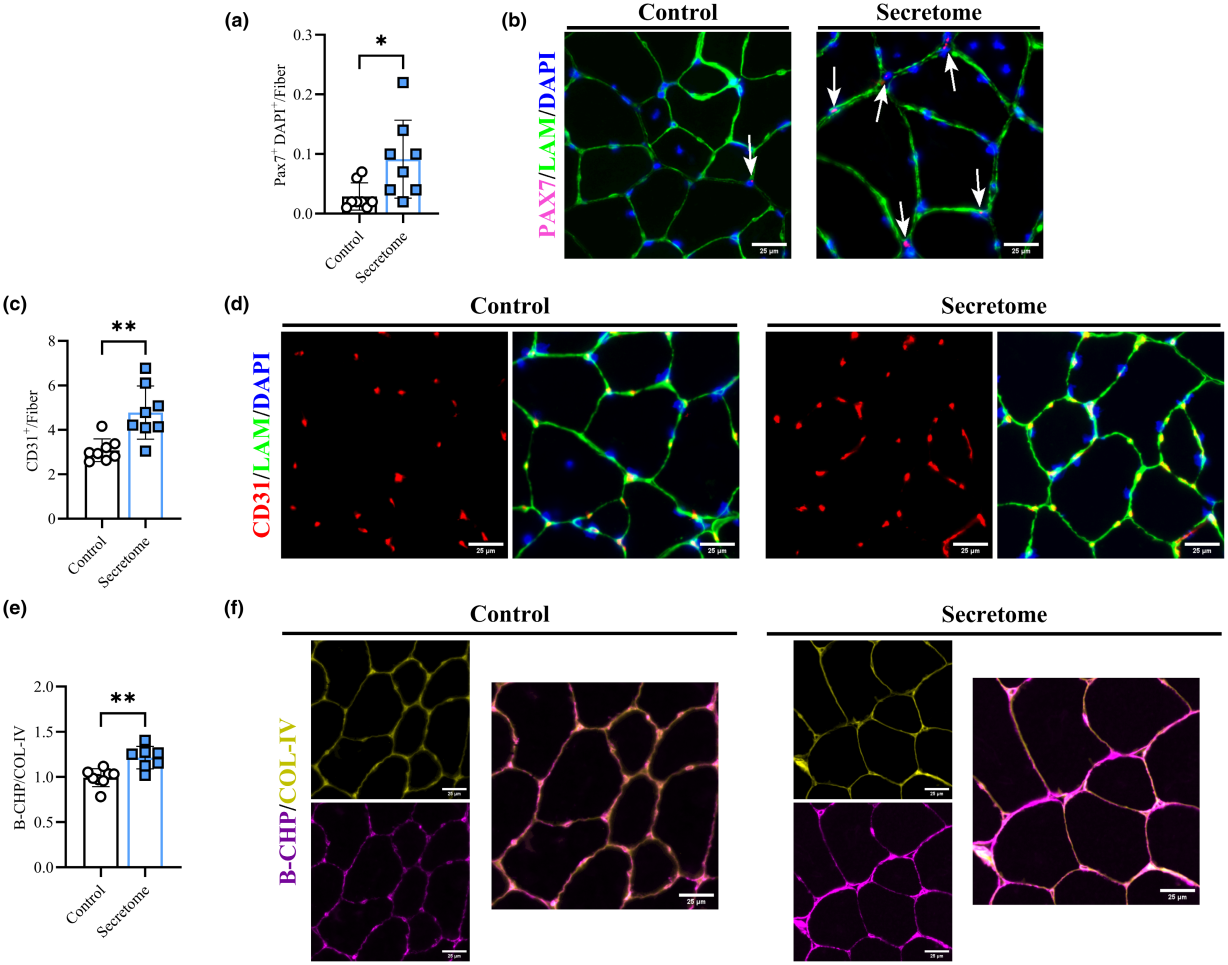
Skeletal Muscle Remodeling. Muscle satellite cell content (Pax7^+^/DAPI^+^ − co‐localization) corrected to number of fibers (a). Representative image of satellite cell localization with PAX7^+^ in off‐red/pink, laminin fiber borders in green, DAPI in blue, satellite cells indicate by white arrows, and scale bar of 50 μm^2^ (b). Fiber capillarization (CD31^+^) corrected to number of muscle fibers (c). Representative image of capillarization with CD31^+^ in red, laminin in green, and DAPI in blue (d). Ratio of B‐CHP to COL‐IV (e) and representative image with B‐CHP in purple and COL‐IV in yellow (f). All data presented as mean ± SD, white circles and bars represent controls while blue squares and bars represent secretome‐treated mice. Control mice (*n* = 8), secretome‐treated mice (*n* = 7–8), right (injected) quadriceps assessed. Analyzed via *t*‐tests. * indicates significant difference between conditions for the indicated timepoints with * = *p* < 0.05, ** = *p* < 0.01.

In parallel with changes to muscle morphology, secretome treatment improved cellular remodeling events in the injected quadricep muscle, affecting satellite cell content, capillarization, and collagen IV turnover. Specifically, compared to saline treatment, secretome‐treated quadriceps showed greater satellite cell content per fiber (Pax7^+^; 0.09 ± 0.06 vs. 0.02 ± 0.02 cells/fiber, *p* = 0.02; Figure 4a) and greater absolute satellite cell count (corrected to total fiber area) (data not shown). In addition, secretome‐treated muscles displayed greater capillary to fiber content (number of CD31^+^ per muscle fibers; Figure 4c; 4.7 ± 1.2 vs. 3.1 ± 0.5 CD31^+^/fiber, *p* < 0.01) as well as greater total CD31^+^ area and CD31^+^ area:fiber ratio (data not shown). Finally, using the ratio of collagen hybridizing peptide (B‐CHP) to collagen IV (COL‐IV) as an indicator of collagen turnover, secretome‐treated muscles had greater Col‐IV turnover (Figure 4e; 1.21 ± 0.1 vs. 0.99 ± 0.1, *p* < 0.01). This effect was the result of greater B‐CHP and lower COL‐IV content in secretome‐treated muscles, which were not significantly different on their own.

As a follow‐up to the 4‐week treatment, we performed a single unilateral right quadriceps injection with the secretome product or saline in a subset (*n* = 10) of aged male C57BL/6 mice (26–28 months old) for analysis of acute transcriptional responses in muscle. After 3 h, injected quadriceps were harvested for bulk RNA sequencing and pathway analysis (Hallmark, KEGG, Reactome). Consistent with the above observed whole body and cellular remodeling events, muscle transcriptomic analysis revealed increases in hypertrophy and mitochondrial‐related pathway enrichment as well as downregulation in inflammatory and immune‐related responses following acute secretome injection in comparison to controls (Figure S2A–C). We additionally conducted western blotting on notable anabolic and catabolic protein phosphorylation events following acute injection of the secretome and found that secretome‐treated mice had increased 4E‐BP1 phosphorylation compared to control mice (1.13 ± 0.09 vs. 1.0 ± 0.12, *p* = 0.01). However, other relevant protein targets (p‐mTOR, p‐rps6k, p‐SMAD2/3, p‐ERK1/2, p‐FOXO3a, and PGC‐1α) were not significantly different between groups (Figure S2D,E). Assessment of a series of proteolytic and atrophy‐related gene targets as reported elsewhere (Palla et al., 2021) by qPCR revealed lower expression levels of E3 ubiquitin ligases (MUSA1, Traf6, FBXO32) in the secretome‐treated group (Figure S2F). Interestingly, at the 4‐week timepoint these transcriptional responses were not different between groups suggesting a transient activation following secretome treatment (Figure S2G).

### 3.4 Intramuscular secretome treatment reduced adiposity in a depot‐specific manner in old mice

**FIGURE 5:**
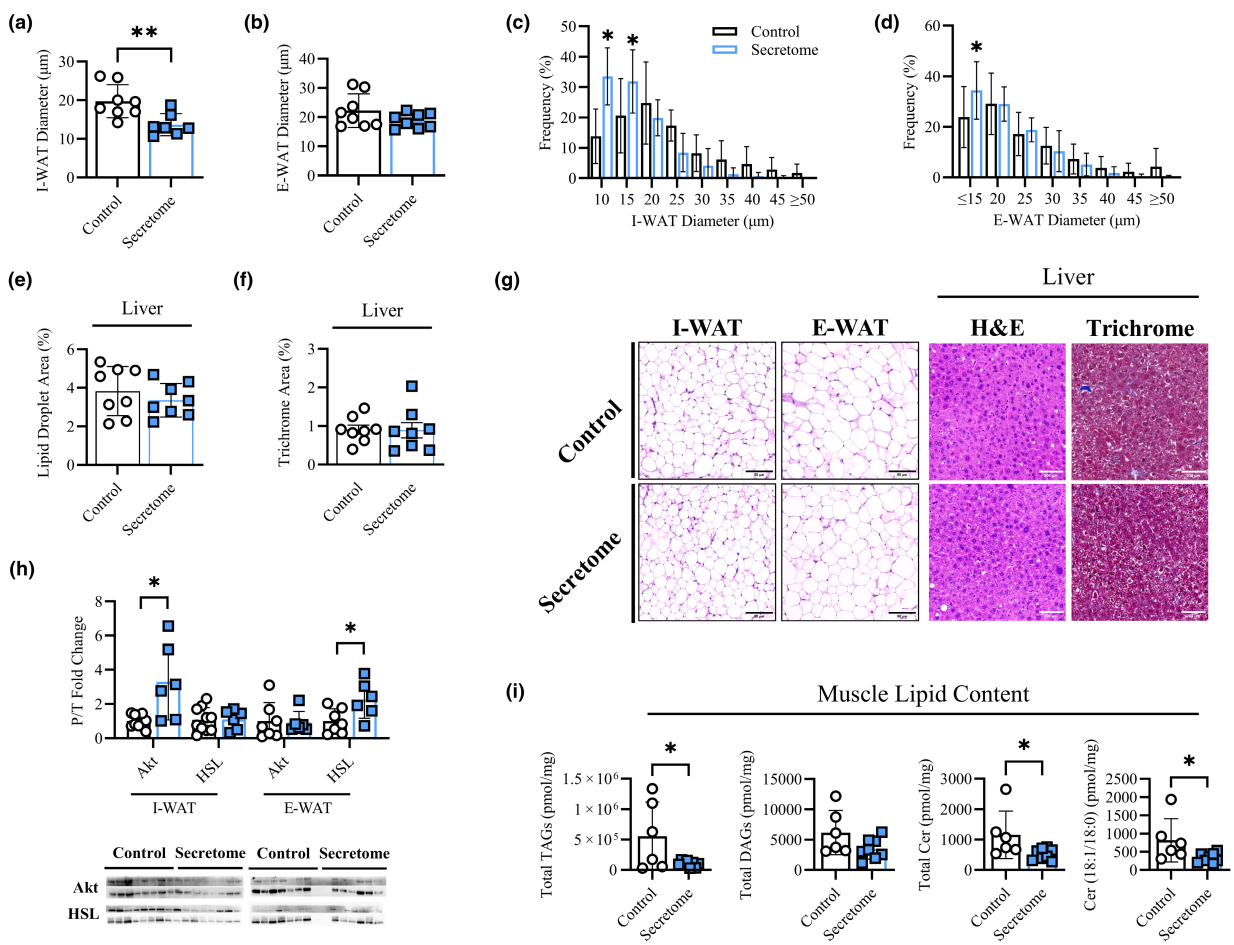
Adipose Morphology and Muscle Lipid Content. Average cellular diameter μm of I‐WAT (a) and E‐WAT (b) depots. Size distribution (%) of I‐WAT (c) and E‐WAT (d) cells across 5 μm increments. Average liver lipid droplet (e) and fibrosis (f; trichrome staining) area (%) as assessed with H&E. Representative image of I‐WAT and E‐WAT depots as well as liver H&E and trichrome staining with scale bar 50 μm (g). Protein phosphorylation status for protein kinase B (Akt) and hormone sensitive lipase (HSL) for I‐WAT and E‐WAT depots and representative western blot image (h). Muscle lipid content including total triglycerides (TAGs) diglycerides (DAGs), ceramides (Cer), and C18:0 ceramide (C18:0 Cer). All data presented as mean ± SD, white circles and bars represent controls while blue squares and bars represent secretome treated groups. (a–g) *n* = 8 for each group, (h–i) *n* = 7 for control, *n* = 6 for secretome, (i) *n* = 6 for control, *n* = 8 for secretome. Analyzed via *t*‐tests (a, b, e, f, h, i) or two‐way ANOVA with Holm‐Bonferroni comparison (c, d). * indicates significant difference between groups at indicated category with * = *p* < 0.05, ** = *p* < 0.01.

As our analysis revealed robust secretome treatment effects on whole body adiposity, we next investigated the localized effects on adipose compartments, namely I‐WAT and E‐WAT depots following 4 weeks of treatment. We found that average I‐WAT diameter (Figure 5a; 13.8 ± 2.8 vs. 19.8 ± 4.3 μm, *p* < 0.01) was lower in secretome‐treated mice while average E‐WAT diameter was not different between groups (Figure 5b). Assessment of adipocyte size distribution in I‐WAT and E‐WAT depots demonstrated a leftward shift in secretome‐treated mice compared to controls. Specifically, there were a greater proportion of smaller adipocytes in the I‐WAT and E‐WAT depots such that I‐WAT cells (Figure 5c) sized 1–10 μm (33.5 ± 8.7 vs. 13.8 ± 8.4%, *p* < 0.01) and 10–15 μm (31.9 ± 9.7 vs. 20.6 ± 11.4%, *p* = 0.02) and the proportion of E‐WAT cells (Figure 5d) ≤15 μm (34.3 ± 10.6 vs. 11.3 ± 11.3%, *p* = 0.03) were higher in secretome‐treated mice compared to control mice. We further assessed protein phosphorylation in both adipose depots and found that secretome‐treated mice had increased I‐WAT Akt phosphorylation (2.7 ± 2.1 vs. 1.0 ± 0.40, *p* = 0.04) and E‐WAT hormone‐sensitive lipase (HSL) (2.3 ± 1.1 vs. 1.0 ± 0.7, *p* = 0.03) phosphorylation compared to controls (Figure 5h). There were no differences in liver lipid accumulation (droplet area) or fibrosis (trichrome staining) between groups (Figure 5e,f). Finally, lipidomic analysis of 4‐week‐treated quadriceps muscle tissue revealed a significant decrease in total intramuscular triglycerides (1.0 × 10^5^ ± 5.5 × 10^5^ vs. 5.5 × 10^5^ ± 4.9 × 10^5^ pmol/mg, *p* = 0.04) and ceramides (519.1 ± 189.5 vs. 1152 ± 782.8 pmol/mg, *p* = 0.04) as well as the muscle‐enriched C18:0 ceramide (334.7 ± 128.6 vs. 813.3 ± 591.9 pmol/mg, *p* = 0.04) in secretome‐treated muscle. Diglyceride content was not different (3494 ± 1693 vs. 6164 ± 3651 pmol/mg, *p* = 0.09) following secretome treatment compared to control mice (Figure 5i).

### 3.5 Secretome treatment altered muscle and adipocyte cells in‐vitro through direct and indirect mechanisms

**FIGURE 6:**
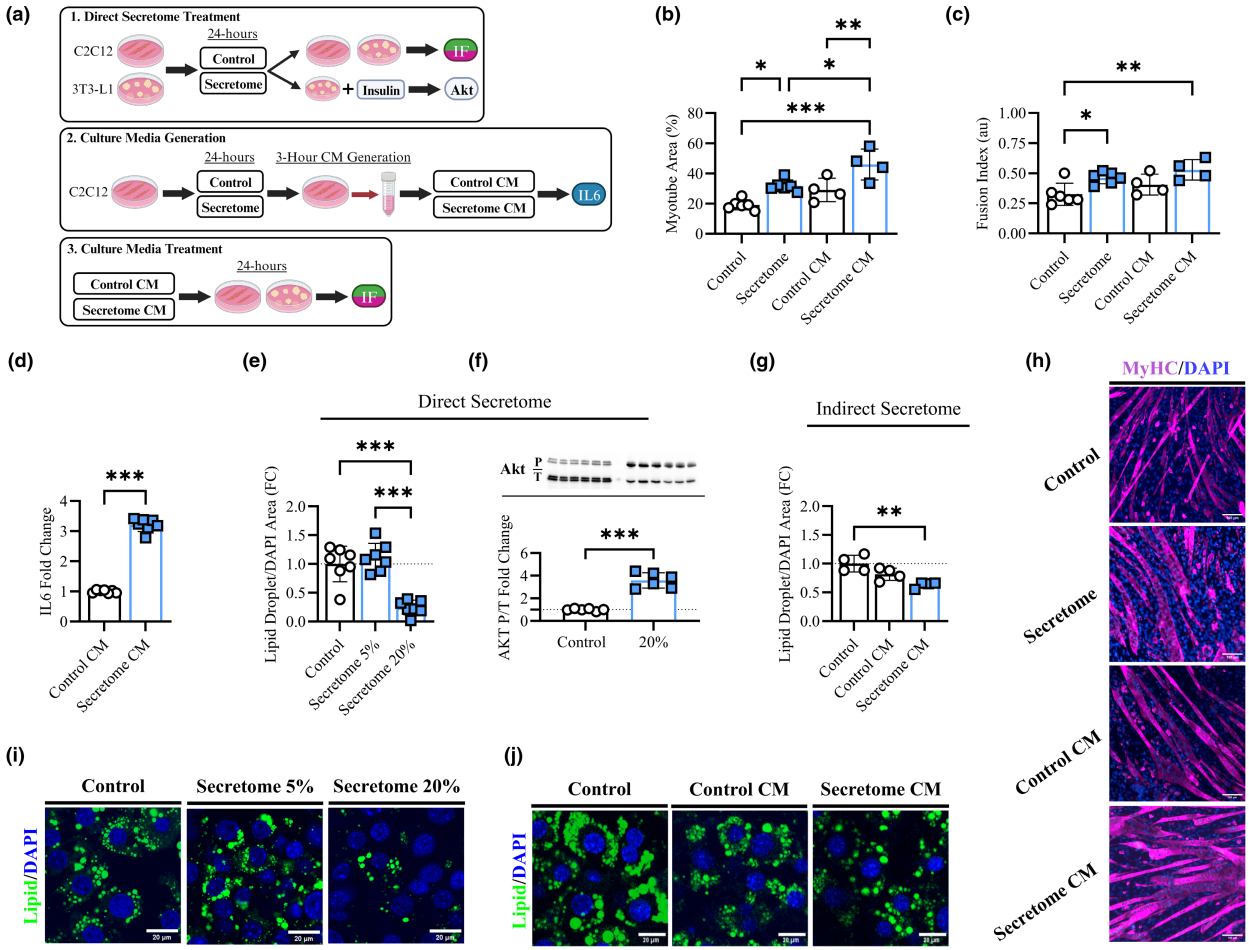
Direct and Indirect Cellular Experiments. Cell culture design for media replacement and cultured media (CM) experiments (a). Average myotube area (%) (b) and myonuclear fusion index (*au*) (c) for control, secretome treated, control CM, and secretome CM conditions in differentiated C2C12 myotubes. Interleukin 6 (IL‐6) content in the culture media collected from secretome treated (4%) and control C2C12 myotubes (Fold change) (d). Average lipid droplet area corrected to DAPI area (Fold Change) in 3T3‐L1 adipocytes for control, 5 and 20% secretome product media replacement (e). Phosphorylated corrected to total Akt protein (fold change) for 20% secretome treated and control 3T3‐L1 adipocytes following overnight fast and insulin (100 nM) stimulation (f). Average lipid droplet area corrected to DAPI area (Fold Change) in 3T3‐L1 adipocytes for control and 20% media replacement with culture media from control and secretome treated C2C12 cells (g). Representative images of myotubes (h) and adipocytes treated with secretome (i) and culture media (j). (b–g) *n* = 4–7 per group or replicate. Analyzed via one‐way ANOVA with Holm‐Bonferroni comparison (b–c, e, g) or *t*‐tests (d, f). * indicates significant difference between groups as indicated with * = *p* < 0.05, ** = *p* < 0.01, *** = *p* < 0.001.

To explore the direct and indirect effects of the secretome product on muscle and adipose tissue, we performed a series of experiments using myotubes and 3T3‐L1 preadipocytes in‐vitro (Figure 6a). Based on our previous publication (Fix et al., 2021), we first confirmed that 4% media replacement with secretome product for 24 h increased myotube area (31.8 ± 3.8 vs. 19.2 ± 3.5%, *p* = 0.01) and myonuclear fusion index (0.46 ± 0.04 vs. 0.32 ± 0.09 *au*, *p* = 0.03) compared to untreated controls (Figure 6b,c). To examine the indirect effects of muscle secretome treatment, we treated a separate group of differentiated myotubes with 4% secretome replacement for 24 h, then allowed them to produce fresh culture media for 3 h (Secretome CM). This cultured media was tested for IL‐6 content, and then the Secretome CM was used to treat both fully differentiated myotubes and adipocytes for 24 h followed by the same analyses as described previously (Figure 6a). IL‐6 was present in control culture media (Control CM) (132.08 ± 7.5 pg/mL) but was over three times as concentrated in Secretome CM (424.02 ± 29.29 pg/mL, *p* < 0.01) (Figure 6d). We additionally tested the undiluted secretome product (*n* = 3) and found considerably lower IL‐6 concentrations (9.86 ± 2.26 pg/mL) confirming that the increased IL‐6 in Secretome CM originated from muscle cells. Compared to Control CM, culture media from secretome‐treated C2C12 myotubes (Secretome CM) increased myotube growth (Figure 6b; 45.93 ± 10.1 vs. 29.06 ± 7.7%, *p* < 0.01) but not fusion index (Figure 6c; 0.52 ± 0.08 vs. 0.40 ± 0.08 *au*, *p* = 0.16). However, Secretome CM increased myotube area over standard control conditions (control) (*p* < 0.001; Figure 6b) and direct secretome treatment (Secretome) (*p* < 0.05) (Figure 6b), and increased fusion index over standard control conditions (*p* < 0.01; Figure 6c). Representative images for myotubes are presented in Figure 6h.

Next, we performed 24 h direct secretome treatment in 3T3‐L1 cells using a low (5%) and high (20%) proportion of media replacement. 20% secretome replacement (but not 5% replacement) robustly decreased the amount of area occupied by lipids corrected for the area of DAPI (Figure 6e; 0.24 ± 0.13 fold change, *p* < 0.01) compared to untreated controls. The reported effects were the same when lipid area was corrected for the area occupied by cell body or number of DAPI (data not shown). Furthermore, 20% secretome replacement decreased the number of lipid droplets per cell corrected to DAPI count (0.31 ± 0.11 fold change, *p* < 0.01) and the average size of lipid droplets (1.9 ± 0.5 vs. 2.7 ± 0.6 μm, *p* = 0.02) compared to control cells (data not shown). In addition, 3T3‐L1 cells treated with 20% secretome replacement displayed enhanced insulin sensitivity as indicated by greater insulin‐stimulated Akt phosphorylation (Ser473) compared to untreated controls (Figure 6f; 3.58 ± 0.67 vs. 1.00 ± 0.10 fold change, *p* < 0.01). Finally, we tested the indirect effects of secretome treatment in 3T3‐L1 adipocytes by replacing 20% of media with the media produced by C2C12 myotubes (i.e., Control CM, Secretome CM), as described above (Figure 6a). Culture media from secretome treated C2C12 myotubes (Secretome CM) reduced lipid area (Figure 6g; 0.63 ± 0.5 fold change, *p* < 0.01) while Control CM did not (Figure 6g; 0.82 ± 0.11 fold change, *p* = 0.11). Secretome‐treated C2C12 culture media (Secretome CM) did not significantly reduce the number of lipid droplets (0.84 ± 0.15 fold change, *p* = 0.15). Preliminary testing with 1% and 5% culture media replacement with Secretome CM had no effect on 3T3‐L1 outcomes regardless of condition (data not shown). Representative images for direct and indirect 3T3‐L1 experiments are presented in Figure 6i,j, respectively.

## 4 DISCUSSION

Here we examined the effects of biweekly intramuscular treatment in aged mice for 4 weeks with a stem cell‐derived secretome product that is enriched with extracellular vesicles and matrix, immunomodulatory, and growth factors. We found that, in 4 weeks, secretome treatment increased lean mass and enhanced local muscle cellular remodeling including reductions in intramuscular lipid content. Remarkably, the secretome treatment enhanced whole‐body energy expenditure, physical activity, and physical function while reducing whole‐body and local adiposity. Moreover, direct secretome treatment in‐vitro robustly enhanced muscle cell growth and reduced lipid droplet content. Finally, cultured media from secretome‐treated muscle cells displayed autocrine and paracrine functions stimulating muscle cell growth and lipid droplet reduction in naive cells. Together, 4‐weeks administration of a stem cell secretome ameliorated several hallmarks of aging in mice resulting in improvements in physical function, metabolic rate, adiposity, and muscle cellular remodeling.

A major finding of this current investigation was that intramuscular secretome treatment in aged mice decreased whole‐body adiposity, reduced fat depot mass and adipocyte size, increased insulin sensitivity and lipolysis (Akt and HSL phosphorylation) in adipose tissues, and improved whole‐body metabolic rate. As adverse body fat accumulates with age (Goodpaster et al., 2005; Wang et al., 2022), the reduction of fat mass following the secretome treatment and the maintenance of this loss after treatment cessation are impactful. Excessive adiposity with age contributes to reduced capacity to perform or adapt to physical activity (Peterson et al., 2011; Phillips et al., 2017; Pontzer et al., 2021), which was also improved following secretome treatment. Previous research demonstrated that 4‐weeks of daily treadmill running modestly increased metabolic rate (VCO_2_ and RER) in old mice while decreasing body fat to levels in young counterparts (Yoon et al., 2021). Accordingly, the robust increases in metabolic outcomes (VO_2_, VCO_2_, RER, activity levels) and reductions in whole‐body and localized fat depots (I‐WAT, E‐WAT) with secretome treatment alone across a similar timeframe as exercise are very promising.

We also report increased whole‐body lean mass as well as greater quadriceps mass and myofiber size, with an emphasis on type II fiber growth, following 4‐weeks of unilateral intramuscular secretome treatment. Additionally, grip strength progressively increased over time and persisted with two weeks of withdrawal independent of lean mass maintenance. These responses were underpinned by robust muscular remodeling including greater collagen IV turnover, capillarization, and muscle stem (satellite) cell content, which are known to enhance muscular function, regeneration, and recovery from disuse atrophy (Fix et al., 2021; Sambasivan et al., 2011). Furthermore, the adaptations to muscle mass and fiber type with secretome treatment are important for maintaining body composition, metabolism (Akasaki et al., 2014), and muscle function with age (Korhonen et al., 2006), and perhaps lends some evidence to the observed improvements to whole‐body metabolic and grip strength. Cumulatively, the rapid increase of muscle size, strength, and remodeling following secretome‐based treatment could be relevant for at‐risk aging populations including those affected by health conditions including metabolic dysfunction and sarcopenia (Fried et al., 2021; Kennedy et al., 2014; Larsson et al., 2019; Pontzer et al., 2021).

Consistent with reduced adiposity and enhanced leanness, we observed lower muscle triglycerides and ceramides following 4‐weeks of secretome treatment. The accumulation of skeletal muscle lipids including triglycerides and ceramides is associated to impaired metabolic health and the development of metabolic diseases (Blachnio‐Zabielska et al., 2016; Turpin‐Nolan et al., 2019). Accordingly, increased intramuscular triglyceride turnover and reductions in storage have positive effects on the health of humans and animals (Bergman et al., 2018; Ko et al., 2018). Improved triglyceride handling is further associated with decreases in muscle ceramides (Bergman et al., 2018), particularly ceramide C18:0, which is linked to insulin resistance and the development of type 2 diabetes (Blachnio‐Zabielska et al., 2016; Turpin‐Nolan et al., 2019). In fact, reducing C18:0 ceramide content in the skeletal muscle of mice improves whole‐body metabolic health (Turpin‐Nolan et al., 2019). Moreover, muscle ceramides have been shown to promote muscle atrophy (De Larichaudy et al., 2012; Morigny et al., 2020) while genetic ablation of ceramides enhanced mitochondrial function and proteostasis in aged mice (Lima et al., 2023). Therefore, reductions in muscle lipids, specifically muscle ceramides, may be a possible mechanism for the improved muscle and physical function following secretome treatment.

The secretome product also enhanced skeletal muscle remodeling in aging noted by higher levels collagen IV turnover, capillarization, and muscle stem cell (satellite cell) content. Interestingly, excessive collagen deposition not only impairs contractile function, but also the ability for satellite cells to proliferate and infiltrate the extracellular muscle environment (Fry et al., 2017; Lacraz et al., 2015). Similarly, greater muscle capillarization, and thereby perfusion, enhances satellite cell dynamics and promotes muscular recovery following damage (Christov et al., 2007), which is diminished yet reversable in aging (Snijders et al., 2017). Accordingly, the robust increase in muscle satellite cell expansion observed here and what we have reported previously (Fix et al., 2021) could be partially driven by enhanced cell migration triggered by enhance perfusion and increased collagen turnover. Noting that satellite cell content and function are limited in aging, an enhanced satellite cell pool would be beneficial to promote muscle regrowth following disuse atrophy (Fix et al., 2021) and/or muscle regeneration following injury (Sambasivan et al., 2011).

The simultaneous adaptations observed for whole‐body and site‐specific lean and fat tissues, as well as whole‐body metabolic rate in the current study are encouraging and warrant discussion. Lean mass is the primary determinant of metabolic rate and is the main site of glucose uptake (Hulett et al., 2022), which may partly explain the greater energy expenditure and glucose utilization (indicated by RER) following secretome treatment in aged mice. Under this premise, a subsequent decrease in fat mass (due to increased lipolysis) may be partially explained by increases in lean mass (and metabolic rate) to mobilize the energy demands of muscle tissue. It is possible that increased physical activity levels in the secretome‐treated mice may further accelerate this cycle. Further, the magnitude and rapidity of effects on body composition observed in the present study are notable and suggestive of direct as well as secondary effects across tissues following secretome treatment.

Appropriately, the secretome product contains a host of bioactive signaling factors (Fix et al., 2021) capable of stimulating growth, metabolism, and remodeling in both muscle and adipose tissue (Hoier & Hellsten, 2014; Zhang et al., 2021). Moreover, we report the presence of extracellular vesicles containing microRNAs within the secretome product, which can have pronounced effects on skeletal muscle, immune cells, and adipose tissues (Kim et al., 2016; Sanz‐Ros et al., 2022; Wu et al., 2022). Though we are not yet able to identify the specific factors driving the direct effects of secretome product treatment, the responses in skeletal muscle and adipose tissue have application to metabolic and musculoskeletal diseases and conditions. Furthermore, while it is unknown if the product directly reaches tissues secondary to the site of injection, we have identified direct and indirect treatment effects in muscle cells and adipocytes with muscle‐derived IL‐6 as a possible mediator for the secondary actions. Both skeletal muscle (Watanabe et al., 2022; Whitham et al., 2018) and adipose tissue (Luo & Liu, 2016; Sjøberg et al., 2017) release extracellular vesicles, myokines, and adipokines upon adequate stimulation, such as exercise. Here, we have shown that prior secretome treatment enhances the production of IL‐6 in myotube culture media, which subsequently stimulates myotube growth and lipid reduction in adipocytes. In agreement, muscle‐derived IL‐6 can mediate adaptations including hypertrophy (Serrano et al., 2008) and lipolysis (Wolsk et al., 2010) in skeletal muscle as well as lipolysis (van Hall et al., 2003) and metabolic reprograming (Li et al., 2021) in adipose tissue. Accordingly, we postulate that secretome treatment may have autocrine and paracrine effects on muscle and fat tissue through the release of IL‐6. However, we cannot rule out other bioactive factors that may be produced by muscle or other cell types following secretome treatment that could be contributing to the phenotype observed in adipose tissue. Future investigations including exploration of tissue‐secreted factors and crosstalk following secretome treatment are justified.

In summary, the results from this experiment show that 4‐weeks of bi‐weekly intramuscular treatment with a stem cell‐derived secretome product enhanced metabolic rate while reducing whole‐body and site‐specific adiposity in aged mice. Moreover, the secretome treatment promoted skeletal muscle hypertrophy, cellular remodeling, and greater physical function in aged mice. Finally, secretome treatment in‐vitro demonstrates both direct and indirect effects on myotube growth and adipocyte lipolysis. Together, these results suggest that as little as 4 weeks of secretome treatment ameliorated many hallmarks of aging in mice. While currently unknown, the translatability of these results to humans is a topic of interest and is under investigation in a phase 1/2a clinical trial (NCT05211986) currently underway. Considering the beneficial pre‐clinical effects reported here and elsewhere, human investigations using stem cell‐derived secretome products are warranted.

## AUTHOR CONTRIBUTIONS

Experiments and data analysis were conducted by Z.J.F, P.B., N.M.D., J.J.P., E.M.Y, and A.K. Experimental design was conducted by M.J.D. The manuscript was prepared by Z.J.F. and M.J.D. All authors contributed to editing and the approval of the final draft of the manuscript.

## FUNDING INFORMATION

Funding for these experiments was provided by Immunis and partly supported by R01AG076075 (M.J.D.).

## CONFLICT OF INTEREST STATEMENT

H.S.K., T.E.L., G.N., N.C.B., and S.G. are employed by Immunis. M.J.D. serves on the Immunis scientific advisory board.

## Supporting information


Figure S1.

Figure S2.

Appendix S1.

Tables S1–S2.

## Data Availability

Data that supports the findings of this study is available in the supplementary material of this article. All data will be made available upon request. RNA‐sequencing data can be found on the NIH Gene Expression Omnibus website located with accession code GSE242211.
