# Supplementary figures and images for: Stem cell secretome treatment improves whole‐body metabolism, reduces adiposity, and promotes skeletal muscle function in aged mice
Source: Aging Cell. 2024 Mar 18;23(6):e14144. doi: 10.1111/acel.14144 (PMC11296109; doi:10.1111/acel.14144)

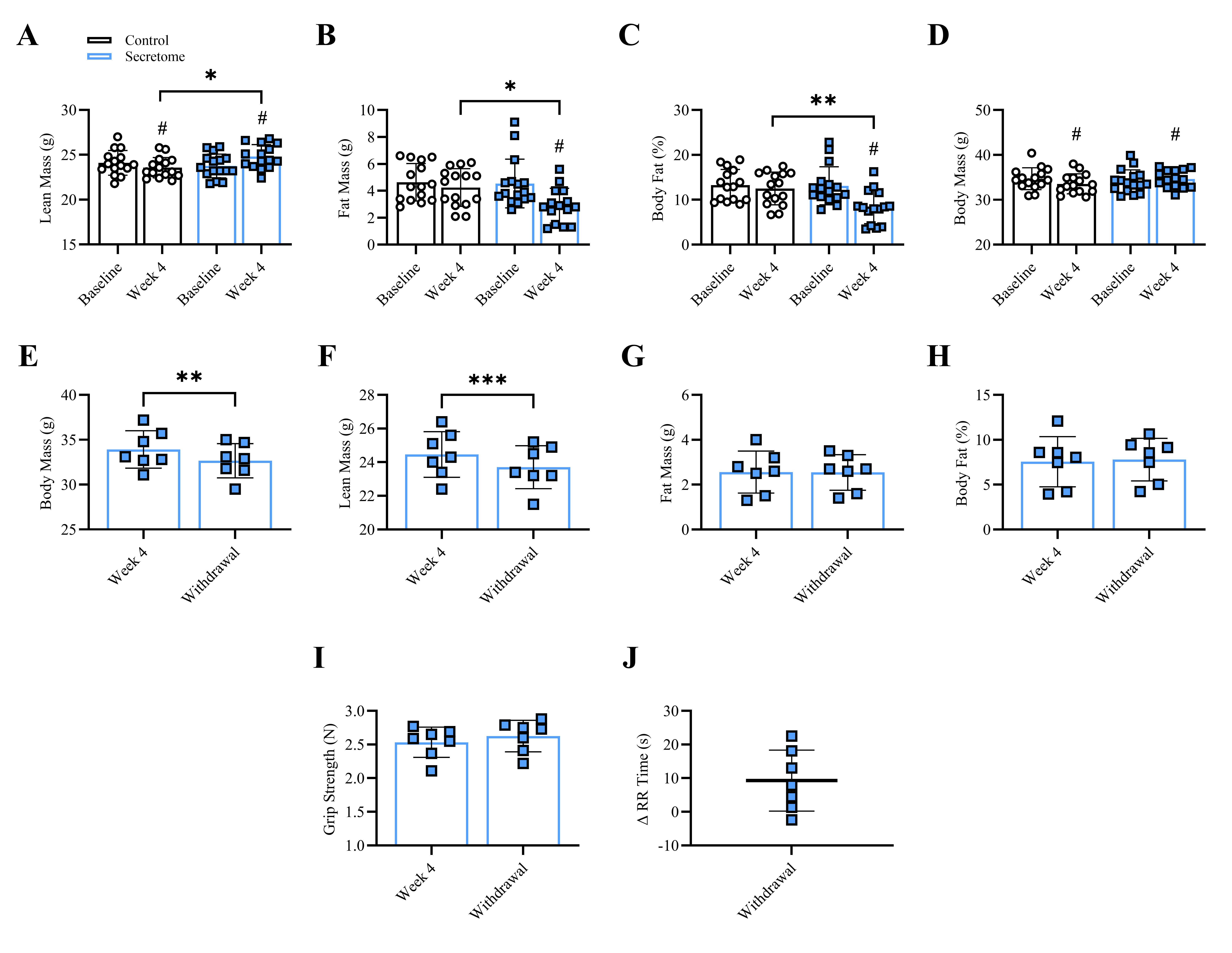

Supplement: Supplementary file 1 — Figure S1. [file ACEL-23-e14144-s004.tif]

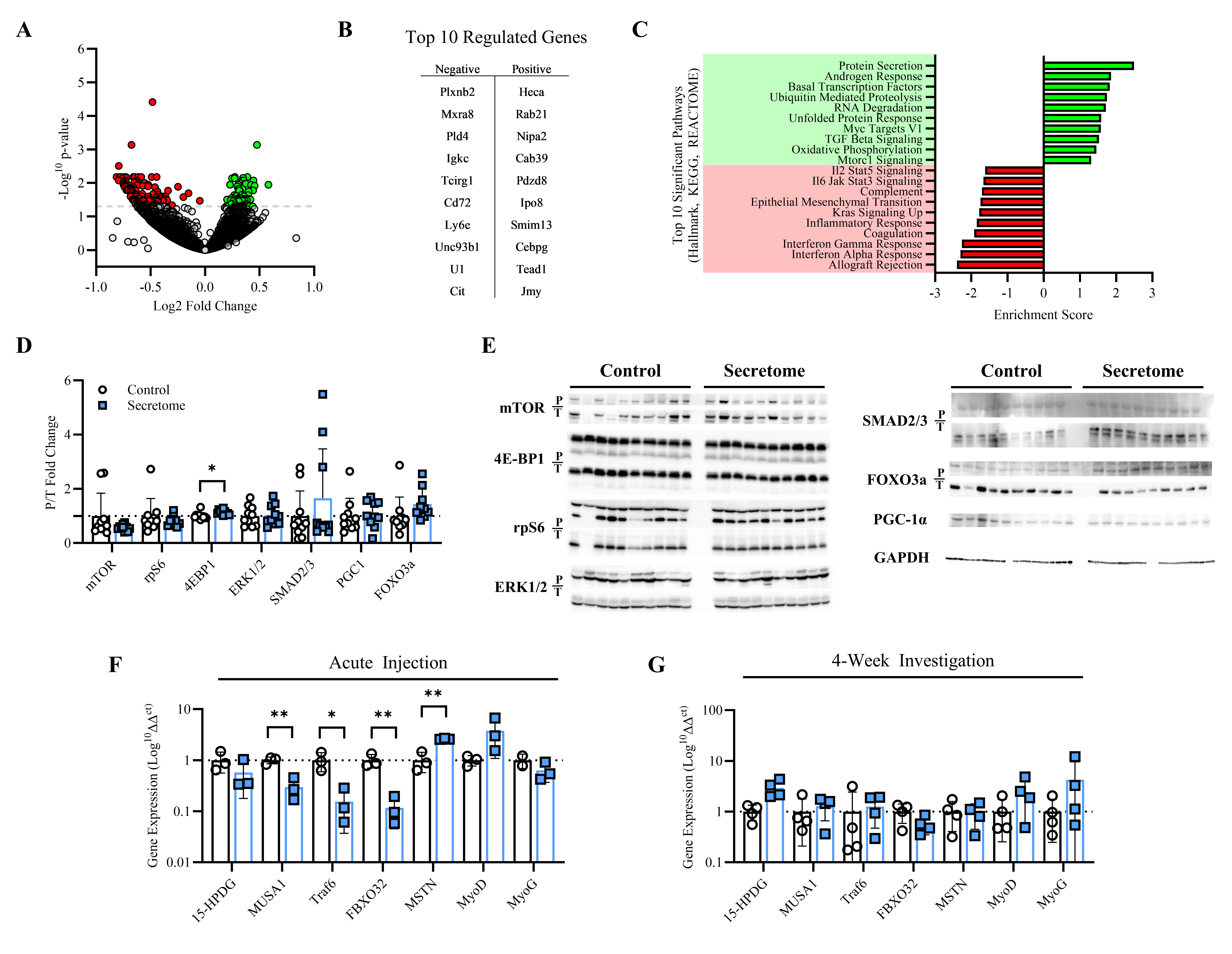

Supplement: Supplementary file 2 — Figure S2. [file ACEL-23-e14144-s002.tif]
