# Supplementary material for: Stem cell secretome treatment improves whole‐body metabolism, reduces adiposity, and promotes skeletal muscle function in aged mice
Source: Aging Cell. 2024 Mar 18;23(6):e14144. doi: 10.1111/acel.14144 (PMC11296109; doi:10.1111/acel.14144)
Supplement: Supplementary file 3 — Appendix S1. [file ACEL-23-e14144-s001.docx]

**Supplemental Methods**

**Bulk RNA Sequencing**

Total RNA was isolated by homogenizing approximately 25 mg of tissue in Qiazol Lysis Reagent (Qiagen, Hilden, DE 79306). The RNA was separated and precipitated using chloroform and isopropanol. Extracted RNA was washed with ethanol then suspended in nuclease-free water. RNA concentration was determined using an EPOCH spectrophotometer (Take3, BioTek, Winooski, VT). Libraries were prepared with Illumina Stranded Total RNA Library Prep NEB Ultra II directional RNA library prep with rRNA depletion and RNA was sequenced using Illumina NovaSeq S4 Reagent Kit v1.5 150x150 bp Sequencing (100 M read-pairs). Differentially expressed genes were identified using a 5% false discovery rate with DESeq2 version 1.30.00 and the hciR package. Fold change cutoff values were set to 1.5 (log2) and significance to 1.3 (-log10).

**Lipid Extraction**

~15 mg of frozen gastrocnemius was homogenized in 225 μL methanol plus an internal standard and 188 μL PBS. A small aliquot of homogenate was collected from each sample for protein quantification and a methanol only process blank was included in the assay. 750μL of methyl tert-butyl ether was added to each sample tube followed by 60min incubation on ice with vortexing every 15min. Samples were centrifuged at 4°C for 10min at 15,000xg and the supernatant containing lipids was transferred to new tubes and evaporated. Lipids were extracted a second time as mentioned above and the organic and aqueous fractions were evaporated and resuspended for subsequent LC-MS metabolomic analysis. All lipid standards were prepared in methanol per manufacturer recommendations (Avanti Polar Lipids, Albaster, AL, USA).

**LC-MC Metabolomics**

Lipid extracts were separated on an Acquity UPLC CSH C18 column (2.1 × 100 mm; 1.7 µm) coupled to an Acquity UPLC CSH C18 VanGuard precolumn (5 × 2.1mm; 1.7µm) (Waters, Milford, MA, USA) maintained at 65°C connected to an Agilent HiP 1290 Sampler, Agilent 1290 Infinity pump, and Agilent 6490 triple quadrupole (QQQ) mass spectrometer. Sphingolipids were detected using dynamic multiple reaction monitoring in positive ion mode. Source gas temperature was set to 175°C, with a gas (N_2_) flow of 15L/min and a nebulizer pressure of 30psi. Sheath gas temperature was 250°C, sheath gas (N_2_) flow of 12L/min, capillary voltage was 3500V, nozzle voltage was 500V, high-pressure RF 190V, and low-pressure RF 120V. Injection volume was 3µL and the samples were analyzed in a randomized order with the pooled QC sample injected 8 times throughout the sample queue. Mobile phase A consisted of ACN:H_2_O (60:40 vol/vol) in 10mM ammonium formate and 0.1% formic acid, and mobile phase B consisted of IPA:ACN: H_2_O (90:9:1 vol/vol/v) in 10mM ammonium formate and 0.1% formic acid. The chromatography gradient started at 15% mobile phase B, increased to 30% B over 0.7 min, increased to 60% B from 0.7 to 1.4min, increased to 80% B from 1.4 to 7.0min, and increased to 99% B from 7.0 to 7.14min, where held until 9.45min before returned to starting conditions at 9.8min. Post-time was 3.5 min and the flow rate was 0.4mL/min throughout.

Data were processed using an Agilent MassHunter Workstation equipped with Qualitative and Quantitative software packages. Pooled quality controls and process blanks were injected throughout the sample queue to ensure the reliability of acquired lipidomics data. Data exported from MassHunter Quantitative analysis was evaluated using Excel where initial lipid targets were parsed based on a relative standard deviation less than 30% in quality control samples. Additionally, only lipids with background counts in process blanks that were less than 30% of the quality controls were used for data analysis. The parsed excel data tables were normalized based on the ratio to class-specific internal standards, then to sample protein concentration.

**Real-Time PCR**

RNA was isolated using Qiazol reagent (Qiagen) with chloroform and isopropanol extraction per manufacturer recommendations and resuspended in nuclease free water. 1 µg of RNA (EPOCH, TAKE3 BioTek) was used to reverse transcribe 20 µl cDNA (iScript cDNA, Bio-Rad) in a thermocycler using the following protocol [25°C 5min, 46°C 20min, 95°C 1min, 4°C (T100, Bio-Rad)]. Real-time PCR was performed with cDNA (1:8 in nuclease-free water) with SsoAdvanced Universal SYBR Green Supermix (Bio-Rad) in a PCR system (CXF Connect, Bio-Rad). All data were normalized to ribosomal protein L32 gene expression, quantified using the delta-delta Ct (2^-ΔΔCt^) method considering fold change from relevant controls, and presented in log^10^. The following primers generated at the University of Utah DNA/Peptide Synthesis core or Bio-Rad (PrimePCR) were used:

| **Primer** | **Catalog #** | **Forward (5’-3’)/Reverse (3’-5’)** |
| --- | --- | --- |
| L32 |  | TTCCTGGTCCACAATGTCAA/  GGCTTTTCGGTTCTTAGAGGA |
| 15-HPDG |  | TCCAGTGTGATGTGGCTGAC/  ATTGTTCACGCCTGCATTGT |
| MUSA1 (FBXO30) |  | TAGTAAGGCTGTTGGAGCTGATAG/  CTGCACCAGTGTGCATAAGG |
| Traf6 (TNFR) |  | TGCAAAAGATGGAACTGAGACATC/  TGGGACAATCCTCAATAATGTGTG |
| FBXO32 (MAFbx) | qMmuCED0045679 |  |
| MSTN | qMmuCED0045853 |  |
| MyoD | qMmuCED0003826 |  |
| MyoG | qMmuCED0001043 |  |

**Western Blotting**

Proteins were isolated in ice-cold lysis buffer [50 mM Tris-HCl pH 7.5, 150 mM NaCl, 5 mM EDTA, 1% Triton X-100, 0.1% sodium deoxycholate, 0.1% SDS, 1X protease and phosphatase inhibitor (Halt, ThermoFisher, Waltam, MA, USA)]. Supernatants were collected following centrifugation at 12,000 g for 15 min at 4°C. Protein concentrations were determined via assay per manufacturer recommendations (Pierce BCA, ThermoScientific). Proteins were loaded equally (20-40ug) and separated by electrophoresis using polyacrylamide gels (4-20%), then transferred to polyvinylidene difluoride membranes (Bio-Rad, Hercules, CA, USA). Ponceau S (K793, VWR, Randor, PA, USA) staining was used to visually confirm protein transfer efficiency. Membranes were blocked for 1 hr in 5% BSA-TSBT at room temperature followed by 3x washes and overnight incubation at 4°C with primary antibodies at1:1000 in 5% BSA-TBST. Following 3x washes, membranes were incubated with secondary antibodies (Cell Signaling anti-mouse 7076 and anti-rabbit 7074, CST, Danvers, MA, USA) at 1:2000 in 5% BSA-TBST for 1 hr at room temperature. Membranes were briefly incubated with ECL Prime Western Blotting Detection Reagent (RPN2236, GE Healthcare, Chicago, IL, USA) then imaged using a ChemiDoc Imaging System (Bio-Rad) and quantified with Image Lab Software (Bio-Rad). Individual proteins were corrected to background and loading controls (GAPDH or Ponceau S) while phosphorylated proteins were corrected to total and expressed as a ratio. All values were expressed as fold change compared to relevant control groups. The following primary antibodies from Cell Signaling were utilized: mTOR, #2983, p-mTOR (ser2448), #2971, 4E-BP1 #9452, p-4E-BP1 (Thr32/46) #2855, rpS6 #2217, p-rpS6 (ser240/244) #2215, SMAD2/3 #3102, ERK1/2 #9102, p-ERK1/2 (Thr202/Tyr204) #9101, p-SMAD2/3 (Ser465/467, Ser423/425) #8828, FOXO3a #2497, p-FOXO3a (Ser253) #9466, Akt #9272, p-Akt (Ser473) #9271, HSL #4107, p-HSL (Ser660) #45804, GAPDH #2118.

**Immunohistochemistry**

Frozen OCT-embedded quadriceps were cut in a longitudinal plane and the anatomically lateral portion were sectioned at a thickness of 10μm using a Leica cryostat (CM1860, Leica, Wetzlar, Hesse, Germany). To assess myofiber cross-sectional area (CSA) and myosin heavy chain (MyHC) fiber type as done before (Petrocelli et al., 2021), sections were blocked in Mouse on Mouse (M.O.M.) (MKB-2212-1, Vector Laboratories, Newark, CA, USA) for 1h. Sections were then incubated in primary antibodies; laminin at a concentration of 1:200 (L9393, Sigma-Aldrich, St. Louis, MO, USA), MyHC I 1:100 (DSHB, BA-D5), MyHC IIa 1:100 (SC-71, DSHB, Iowa City, IA, USA), MyHC IIb 1:100 (BF-F3, DSHB); in 2.5% horse serum (160501-30, Gibco, Billings, MT, USA) (in 1x PBS) overnight. Next, slides were washed and incubated in secondary antibodies; AMCA 1:500 (CI-1000, Vector Laboratories), MyHC I 1:250 (A21242, Invitrogen, Carlsbad, CA, USA), MyHC IIa 1:500 (A21121, Invitrogen), MyHC IIb 1:500 (A21426, Invitrogen); in 1x PBS for 1h. A similar number of fibers were analyzed for control and secretome treated quadriceps (2827 ± 813 vs 2547 ± 828, p=0.50). For satellite cell/muscle stem cell content, muscle sections were fixed in 4% paraformaldehyde, washed, and heated in sodium citrate buffer from 65-92°C for 20 min and cooled to room temperature for antigen retrieval. Endogenous peroxidases were blocked with 3% H_2_O_2_ followed by 1h in M.O.M. IgG blocker (MKB-2213, Vector, San Diego, CA, USA ) and 1h in 1% BSA (in 1x PBS). Muscles were incubated in primary PAX7 antibody 1:100 (Pax7c, DSHB) and laminin 1:200 (L9393, Sigma-Aldrich) overnight at 4°C. Following washes, slides were incubated in secondary antibodies for PAX7 1:1000 (15-065-205, Jackson ImmunoResearch, West Grove, PA, USA) and laminin 1:500 (A21429, Invitrogen); in 1% BSA in PBS. The Tyramide SuperBoost Kit with Alexa Fluor 568 (B40956, Invitrogen) was used following the manufacturer’s instructions to amplify the PAX7 signal. Slides were finally incubated for 5 min in DAPI 1:10,000 (D3571, Invitrogen) then washed again. To assess capillarization, muscle sections were fixed in acetone for 10 min at -20°C, washed, then blocked in 3% H2O2 7 min. Next slides were washed and blocked in 2.5% NHS for 1h then washed again. Muscles were incubated in primary CD31 antibody 1:100 (550274, BD Biosciences, Franklin Lakes, NJ, USA) and laminin 1:200 (L9393, Sigma-Aldrich) overnight at 4°C. Following washes, slides were incubated in secondary antibodies for CD31 1:250 (A10522, ThermoFisher) and laminin 1:500 (A21429, Invitrogen) for 1hr in 2.5% NHS. Slides were then washed, incubated in DAPI 1:10,000 (D3571, Invitrogen), then washed again. To visualize collagen turnover (Ferrara et al., 2022; Fix et al., 2021; Petrocelli et al., 2021), we examined the expression of collagen hybridizing peptide, a marker of collagen breakdown (Hwang et al., 2017), to collagen IV, which is prominent in the basement membrane of skeletal muscle (Gelse, Pöschl, & Aigner, 2003). Slides were fixed in acetone for 10 min at -20°C, washed, then blocked in 2.5% NHS for 1 hour. 15 µM biotinylated-collagen hybridizing peptide (B-CHP) was incubated at 80°C for 5 min then quickly cooled in ice water. Slides were incubated in B-CHP (3-Helix, Salt lake City, UT, USA) plus primary antibody for collagen IV (COL-IV) 1:100 (ab6586, Abcam, Cambridge, UK) overnight at 4°C. Slides were washed then incubated in Dylight 1:200 (SA5549, Thermofisher) plus secondary antibody for collagen IV 1:250 (21245, Invirogen) for 1 hr. Following washes, slides were incubated in DAPI 1:10,000 (D3571, Invitrogen), then washed again. All slides were mounted with vector shield fluorescent mounting media (H-1000, Vector, San Diego, CA, USA). Stained sections were imaged on a Zeiss Slide Scanner Axio Scan.Z1 (Carl Zeiss Inc., Oberkochen, DE) at 10x (CSA-MyHC, B-CHP/COL-IV) or 20x (PAX7, CD31) objective. CSA-MyHC and CD31 Images were adjusted for contrast and brightness using Fiji software (v2.9.0) (Schindelin et al., 2012) and analyzed using semi-automatic muscle analysis using segmentation of histology: a MATLAB application (SMASH) as described previously (Smith & Barton, 2014). Satellite cells were counted with Fiji and determined by average co-occurrence with Pax7+ and DAPI+ positive cells for each sample across 3 randomly selected fields. Collagen turnover (B-CHP/COL-IV) was assessed using the Nikon Elements Advanced Research software (Nikon, Tokyo, Japan) as previously described (Petrocelli et al., 2021).

**Cell Culture and Treatment**

C2C12 myoblasts (CRL-1772, ATCC, Manassas, VA, USA) were expanded according to manufacturer recommendations and cultured into differentiated myotubes for experiments. Cells were plated at approximately 5x10^3^ cells/cm^2^ in 6-well dishes (140675, Thermofisher) and grown to confluence for ~6 days in growth medium (DMEM 4.5 g/dL, 10% FBS, 1% penicillin-streptomycin) then differentiated into myotubes for ~4 days in myotube differentiation medium (DMEM 4.5 g/dL, 2% HS, 1% penicillin-streptomycin). For direct secretome experiments, differentiated cells were incubated with fresh myotube differentiation media containing 4% secretome product for 24 hours. Cells were washed 3x in PBS, fixed in ice cold paraformaldehyde (4%) for 30 min, permeabilized in triton x-100 (0.1% in PBS) for 10 min, and washed 3x again. Cells were incubated in with myosin heavy chain 4 conjugated antibodies (MF20, eBioscience, San Diego, CA, USA) for 1 hour (3% HS in PBS) at room temperature followed by 10 min in DAPI at 1:10,000 (D3571, Invitrogen) and 3x washes. Cells were imaged in triplicate (EVOS FL, Invitrogen) and averaged per well across condition replicates. Myotube area was determined as the field of view covered by functional myotubes (3+ nuclei) and fusion index as the number of nuclei contained within functional myotubes compared to total nuclei. Subsequent groups of fully differentiated control and secretome treated cells (4%) were washed 3x and incubated in fresh differentiation media for 3 hours to acquire cultured media. Cultured media was collected and pooled, centrifuged (150xg, 7 min) to remove cellular debris, and frozen at -20°C. Collected C2C12 culture media (n=7) as well as undiluted secretome product (n=3) was assessed for IL-6 concentrations using a Mouse IL-6 Immunoassay (Quantikine ELISA M6000B-1, R & D Systems, Minneapolis, MN, USA) per manufacturer recommendations. Finally, a separate group of differentiated C2C12’s were incubated in the cultured media previously described for 24 hours followed by the same analyses as above.

3T3-L1 fibroblasts (CL-173, ATCC) were expanded according to manufacturer recommendations and cultured into adipocytes for experiments (Montanari, Boschi, & Colitti, 2019). Cells were plated at approximately 3x10^3^ cells/cm^2^ in 6- and 8-well chamber slides (177445, Thermofisher) for 5-7 days until 100% confluent in growth medium followed by 3 days in adipocyte differentiation medium (DMEM/F-12, 10% FBS, 1% penicillin-streptomycin, 1 ug/mL insulin, 0.5 µM dexamethasone, 0.5 mM isobutylmethylxanathine). Media was replaced every 48-72 hours during this period. Following differentiation, cells were kept in maintenance medium (DMEM/F-12, 10% FBS, 1% penicillin-streptomycin, 1 ug/mL insulin) for 24 hours. For direct secretome experiments 5% and 20% of total maintenance media was replaced with the secretome product while maintaining insulin concentration for 24 hours. Following control and secretome treatment conditions, additional groups of adipocytes were maintained in maintenance medium without serum or insulin for ~16 hours. Cells were then spiked with insulin (100 nM) for 15 min and lysed for protein analysis as detailed previously. For indirect secretome experiments, the C2C12 culture media as described above was used to incubate adipocytes for 24 hours at 20% replacement. As indirect experiments were performed 3+ months following initial experiments and required use of completely new reagents and a different microscope of the same model, we repeated untreated controls for normalization. To assess lipid droplets, cells were stained with BODIPY (Thermofisher, D3922). Briefly, cells were rinsed, fixed in 4% PFA for 25 min, washed, then incubated in 2 µM BODIPY for 60 min in the dark. Cells were then washed, incubated in DAPI 1:10,000 for 15 min, and washed again. Cells were mounted (H-1000, Invitrogen), sealed, and imaged at 40x for direct experiments (SP8 Confocal White Light, Leica Microsystems, Wetzlar, DE), and 20x for indirect experiments (SP8 Confocal 4-Channel), Leica Microsystems). Lipid droplets were analyzed with Fiji by thresholding and analyzing area across a minimum of four images per condition replicate. Additionally, lipid droplet count and particle properties were analyzed using ALDQ (Exner et al., 2019) and MorphoLibJ (Legland, Arganda-Carreras, & Andrey, 2016) plugins.

Specific Reagents:

- DMEM 4.5 g/L D-glucose (Thermofisher, 11965-092).
- DMEM/F-12 + GlutaMAX (Thermofisher, 2537548).
- Fetal Bovine Serum (FBS) (Gen Clone, 25-550).
- Penicillin-streptomycin (Thermofisher, 15140122).
- Insulin Solution 10 mg/mL (Sigma, I0516).
- Dexamethasone (Sigma, D4902).
- 3-Isobutyl-1-methylxanathine (Sigma, I5879).

References

Exner, T., Beretta, C. A., Gao, Q., Afting, C., Romero-Brey, I., Bartenschlager, R., . . . Füllekrug, J. (2019). Lipid droplet quantification based on iterative image processing. *J Lipid Res, 60*(7), 1333-1344. doi:10.1194/jlr.D092841

Ferrara, P. J., Yee, E. M., Petrocelli, J. J., Fix, D. K., Hauser, C. T., de Hart, N. M. M. P., . . . Drummond, M. J. (2022). Macrophage immunomodulation accelerates skeletal muscle functional recovery in aged mice following disuse atrophy. *J Appl Physiol (1985), 133*(4), 919-931. doi:10.1152/japplphysiol.00374.2022

Fix, D. K., Ekiz, H. A., Petrocelli, J. J., McKenzie, A. M., Mahmassani, Z. S., O'Connell, R. M., & Drummond, M. J. (2021). Disrupted macrophage metabolic reprogramming in aged soleus muscle during early recovery following disuse atrophy. *Aging Cell, 20*(9), e13448. doi:10.1111/acel.13448

Gelse, K., Pöschl, E., & Aigner, T. (2003). Collagens--structure, function, and biosynthesis. *Adv Drug Deliv Rev, 55*(12), 1531-1546. doi:10.1016/j.addr.2003.08.002

Hwang, J., Huang, Y., Burwell, T. J., Peterson, N. C., Connor, J., Weiss, S. J., . . . Li, Y. (2017). In Situ Imaging of Tissue Remodeling with Collagen Hybridizing Peptides. *ACS Nano, 11*(10), 9825-9835. doi:10.1021/acsnano.7b03150

Legland, D., Arganda-Carreras, I., & Andrey, P. (2016). MorphoLibJ: integrated library and plugins for mathematical morphology with ImageJ. *Bioinformatics, 32*(22), 3532-3534. doi:10.1093/bioinformatics/btw413

Montanari, T., Boschi, F., & Colitti, M. (2019). Comparison of the Effects of Browning-Inducing Capsaicin on Two Murine Adipocyte Models. *Front Physiol, 10*, 1380. doi:10.3389/fphys.2019.01380

Petrocelli, J. J., Mahmassani, Z. S., Fix, D. K., Montgomery, J. A., Reidy, P. T., McKenzie, A. I., . . . Drummond, M. J. (2021). Metformin and leucine increase satellite cells and collagen remodeling during disuse and recovery in aged muscle. *FASEB J, 35*(9), e21862. doi:10.1096/fj.202100883R

Schindelin, J., Arganda-Carreras, I., Frise, E., Kaynig, V., Longair, M., Pietzsch, T., . . . Cardona, A. (2012). Fiji: an open-source platform for biological-image analysis. *Nat Methods, 9*(7), 676-682. doi:10.1038/nmeth.2019

Smith, L. R., & Barton, E. R. (2014). SMASH - semi-automatic muscle analysis using segmentation of histology: a MATLAB application. *Skelet Muscle, 4*, 21. doi:10.1186/2044-5040-4-21
