# Supplementary material for: Stem cell secretome treatment improves whole‐body metabolism, reduces adiposity, and promotes skeletal muscle function in aged mice
Source: Aging Cell. 2024 Mar 18;23(6):e14144. doi: 10.1111/acel.14144 (PMC11296109; doi:10.1111/acel.14144)
Supplement: Supplementary file 4 — Tables S1–S2. [file ACEL-23-e14144-s003.docx]

**Supplemental Table 1.** Tissue Weights

|  | **Quadriceps (mg)** | | | **Gastrocnemius (mg)** | | | **Adipose (mg)** | | | **Secondary Tissue** | | |
| --- | --- | --- | --- | --- | --- | --- | --- | --- | --- | --- | --- | --- |
|  | **Right** | **Left** | **Combined** | **Right** | **Left** | **Combined** | **I-WAT** | **E-WAT** | **Combined** | **Heart (mg)** | **Liver (mg)** | **Femur (*au*)** |
| **Control** | 165.3 ± 16.7 | 169.2 ± 23.7 | 334.5 ± 31.7 | 119.2 ± 14.4 | 139.2 ± 10.2 | 258.4 ± 22.5 | 591.7 ± 152.9 | 772.2 ± 266.1 | 1363.9 ± 323.3 | 168.4 ± 15.0 | 1408.6 ± 190.2 | 17.8 ± 3.2 |
| **Secretome** | 190.1 ± 18.9 | 191.0 ± 15.9 | 379.1 ± 31.1 | 130.4 ± 21.8 | 142.8 ± 16.5 | 273.2 ± 32.4 | 462.0 ± 103.4 | 723.1 ± 273.0 | 1246.9 ± 429.8 | 174.1 ± 29.1 | 1590.3 ± 139.2 | 21.7 ± 6.0 |
| ***T-Test*** | **0.01* | **0.02* | **<0.01* | *0.12* | *0.30* | *0.15* | **0.03* | *0.36* | *0.26* | *0.63* | *0.06* | *0.26* |
| Tissue and organ weights following 4-weeks for control and secretome treated mice. Muscle tissues include right, left, and combined weights for quadriceps and gastrocnemius. Adipose tissues include I-WAT, E-WAT, and combined depots. Heart and liver weights are absolute while femur units indicate the ratio between bone volume and trabecular thickness. All data presented as mean ± SD. n=8 for muscles, adipose, and organs, n=5 for femurs. Analyzed via t-tests. * indicates significant difference between conditions. | | | | | | | | | | | | |

**Supplemental Tables 2.** Tissue Weights Corrected to Body Mass

|  | **Quad. (au)** | | | **Gast. (au)** | | | **Adipose (au)** | | | **Secondary Tissues** |  |
| --- | --- | --- | --- | --- | --- | --- | --- | --- | --- | --- | --- |
|  | **Right** | **Left** | **Combined** | **Right** | **Left** | **Combined** | **I-WAT** | **E-WAT** | **Combined** | **Heart (g)** | **Liver (g)** |
| **Control** | 4.95 ± 0.37 | 5.06 ± 0.51 | 10.01 ± 0.41 | 3.57 ± 0.33 | 4.17 ± 0.14 | 7.74 ± 0.39 | 14.93 ± 6.14 | 22.92 ± 7.21 | 37.85 ± 8.10 | 5.05 ± 0.42 | 42.33 ± 6.12 |
| **Secretome** | 5.16 ± 0.54 | 5.40 ± 0.46 | 10.56 ± 0.76 | 3.70 ± 0.64 | 4.04 ± 0.34 | 7.74 ± 0.85 | 13.08 ± 2.82 | 20.45 ± 7.60 | 33.53 ± 9.21 | 4.92 ± 0.72 | 45.28 ± 3.44 |
| ***T-Test*** | *0.39* | *0.18* | *0.09* | *0.61* | *0.32* | *0.99* | *0.28* | *0.67* | *0.45* | *0.52* | *0.34* |
| Tissue and organ weights following 4-weeks for control and secretome treated mice corrected to body mass. Muscle tissues include right, left, and combined ratios for quadriceps and gastrocnemius. Adipose tissues include I-WAT, E-WAT, and combined depots. Secondary tissues include heart and liver weights. All data presented as mean ± SD. n=8 . Analyzed via t-tests. * indicates significant difference between conditions. | | | | | | | | | | | |
